# The ixabepilone and vandetanib combination shows synergistic activity in docetaxel-resistant MDA-MB-231 breast cancer cells

**DOI:** 10.1007/s43440-022-00396-7

**Published:** 2022-07-30

**Authors:** Stanton Tam, Yassir Al-Zubaidi, Md Khalilur Rahman, Kirsi Bourget, Fanfan Zhou, Michael Murray

**Affiliations:** 1 Pharmacogenomics and Drug Development Group, Discipline of Pharmacology, School of Medical Sciences, University of Sydney, New South Wales, 2006 Australia; 2 Sydney Pharmacy School, Faculty of Medicine and Health, University of Sydney, New South Wales, 2006 Australia; 3 Present Address: College of Pharmacy, The University of Mashreq, Baghdad, Iraq

**Keywords:** Drug resistance, Ixabepilone, EGFR-receptor inhibitors, Combination treatments

## Abstract

**Background:**

The lack of drug targets is an obstacle to the treatment of patients with triple-negative breast cancer (TNBC). At present, non-specific cytotoxic drugs are first-line agents, but the development of resistance is a major problem with these agents. The epidermal growth factor receptor (EGFR) is a potential target in some TNBCs, because its tyrosine kinase activity drives tumorigenesis. Thus, small molecule inhibitors of the EGFR in combination with cytotoxic agents could be important for the treatment of TNBCs.

**Methods:**

The present study evaluated the efficacies of clinically approved EGFR inhibitors in combination with the cytotoxic agent ixabepilone in parental and docetaxel-resistant MDA-MB-231 cells (231C and TXT cells, respectively). Cell viability was assessed using MTT reduction assays, cell death pathways were evaluated using annexin V/7-aminoactinomycin D staining and flow cytometry and Western immunoblotting was used to assess the expression of pro- and anti-apoptotic proteins in cells.

**Results:**

Ixabepilone and the EGFR inhibitors gefitinib and vandetanib inhibited 231C and TXT cell proliferation, but the alternate EGFR inhibitors erlotinib and lapatinib were poorly active. Using combination analysis, ixabepilone/vandetanib was synergistic in both cell types, whereas the ixabepilone/gefitinib combination exhibited antagonism. By flow cytometry, ixabepilone/vandetanib enhanced 231C and TXT cell death over that produced by the single agents and also enhanced caspase-3 cleavage and the pro/anti-apoptotic Bcl-2 protein ratios over ixabepilone alone.

**Conclusions:**

These findings suggest that the ixabepilone/vandetanib combination may have promise for the treatment of patients with drug-resistant TNBC.

**Graphical abstract:**

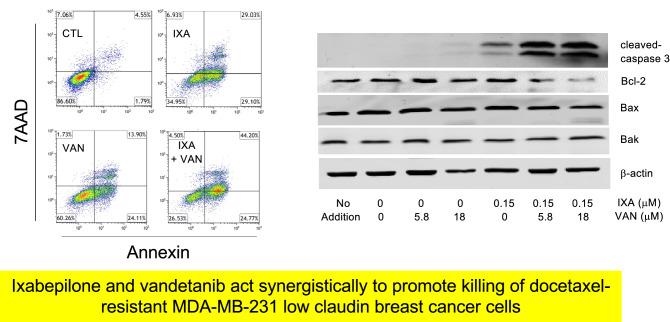

**Supplementary Information:**

The online version contains supplementary material available at 10.1007/s43440-022-00396-7.

## Introduction

Triple-negative breast cancers (TNBCs) are an aggressive and highly heterogeneously group of tumors that represent 10–20% of all breast cancers [1]. From gene profiling, the distribution of subtypes includes basal-like (49%), claudin-low (37%), HER2-enriched (9%), normal-like (4%), and luminal A and B (~ 1%) [2]. The claudin-low subtype in particular is associated with a very poor prognosis and poor response to drug treatment [3].

TNBCs lack the estrogen, progesterone, and HER2 receptors that are expressed in non-TNBC breast cancers and that can be targeted therapeutically [4]. Thus, the current front-line treatments for TNBC are non-targeted cytotoxic agents, including taxanes, such as paclitaxel and docetaxel, anthracyclines, such as doxorubicin, and platinum agents like cisplatin [5, 6]. Although initially effective, most patients on prolonged therapy with these agents suffer relapse. Indeed, prolonged exposure to taxanes upregulates the efflux transporter ABCB1 (P-glycoprotein) that pumps anticancer drugs out of tumor cells [7]. The failure to achieve therapeutic drug concentrations in tumors is an important mechanism of resistance.

In patients with drug-resistant TNBC, treatment must be switched to different drugs or additional medications must be added to the regimen. Combination therapy can improve therapeutic efficacy by targeting multiple tumorigenic mechanisms in synergistic or additive fashion. Although the identification of validated drug targets in TNBC tumors has not yet been achieved, one potential target of interest is the epidermal growth factor receptor (EGFR) that is associated with poor rates of survival in TNBC patients [8, 9]. Indeed, cytotoxic agents in combination with EGFR-targeting antibodies like cetuximab have been found to be effective in TNBC cells in vitro and in some patients with metastatic TNBC [10]. Tyrosine kinase inhibitor (TKI) drugs have revolutionised the treatment of many cancers and TKIs such as gefitinib, erlotinib, and lapatinib that inhibit the EGFR are effective agents in the treatment of certain cancers. The preclinical evaluation of combinations of cytotoxic agents with anti-EGFR TKIs in TNBC cells could identify new approaches that could then be evaluated in clinical trials.

Ixabepilone is a semi-synthetic analogue of epothilone that is approved for use in the treatment of metastatic or locally advanced breast cancer after the failure of front-line agents [11]. Ixabepilone is a substrate for ABCB1, but not alternate efflux transporters such as breast cancer resistance protein (BCRP) [12]. It has been shown that ixabepilone is more active than other cytotoxic agents in taxane-resistant cell lines [13]. Vandetanib is an anti-EGFR TKI that is approved for the treatment of advanced medullary thyroid cancer [14]. Vandetanib is also effective in combination with docetaxel in patients with previously treated non-small-cell lung cancer [15] and, in xenograft studies, promoted the regression of TNBC tumors that expressed EGFR [16]. Vandetanib also inhibits ABCB1, which could enhance the retention of coadministered cytotoxic agents in tumor cells [17].

The present study evaluated the combination of ixabepilone and the EGFR TKI vandetanib in parental and docetaxel-resistant MDA-MB-231 cells (TXT) that are established models of claudin-low TNBC [18]. Comparative studies were also undertaken with gefitinib, which is a more widely used EGFR TKI in patients. The principal findings to emerge were that the ixabepilone/vandetanib combination synergistically decreased the viability of both 231C and TXT cells, whereas the combination of ixabepilone with getifinib exhibited antagonism.

## Materials and methods

### Chemicals and biochemicals

Unless otherwise stated biochemicals were obtained from Sigma-Aldrich (Castle Hill, NSW, Australia). Dulbecco's Modified Eagle's Medium (DMEM), Fetal Bovine Serum (FBS), L-glutamine, trypsin/EDTA, penicillin, and streptomycin and phosphate-buffered saline (PBS) were from Sigma. Reagents for electrophoresis were from Bio-Rad (Richmond, CA) and the annexin V-FITC/7-aminoactinomycin D (7AAD) dye kit was obtained from Beckman Coulter (Gladesville, NSW, Australia). Vandetanib, gefitinib, ixabepilone, lapatinib, erlotinib, and docetaxel were obtained from Selleckchem (Sapphire Bioscience, Redfern, NSW, Australia). General analytical grade laboratory chemicals and HPLC grade solvents were obtained from LabScan (Lomb Scientific, Taren Point, NSW, Australia) or Ajax Chemicals (Sydney, NSW, Australia).

Anti-cleaved caspase-3 (Cat. no. 9661S), anti-Bax (5023S), anti-Bak (12105S), anti-Bcl-2 (2870S), and anti-GAPDH (2118S) were from Cell Signaling Technology (Arundel, QLD, Australia). Alexa fluor-conjugated anti-mouse (4408S), anti-rabbit (4412S) IgG, the Dylight-conjugated goat anti-mouse (5470S), and goat anti-rabbit (5151S) secondary antibodies were also purchased from Cell Signaling Technology.

### Cell viability assay

Human MDA-MB-231-derived 231C and TXT cells were generous gifts from Dr Branimir I. Sikic (Department of Medicine, Stanford University, CA) and were kindly provided by Mr Stephen Miles and Prof Kum Kum Khanna (Queensland Institute for Medical Research, Herston, QLD, Australia). Cells were free from mycoplasma and were maintained in DMEM-high glucose media supplemented with 10% FBS and 1% penicillin/streptomycin.

The reduction of MTT was used to assess cell viability, as described previously [19]. Cells were seeded in triplicate in 96-well plates at a density of 5 × 10^3^/well and incubated overnight (5% CO_2_ and 37 °C). Test drugs were added to cultured cells (in DMSO, 0.1% final); control cells received an equivalent volume of solvent (DMSO) alone for 48 h followed by MTT (62.5 μg/25 μL). Plates were then incubated at 37 °C for 2 h after which 100 μL DMSO was added to dissolve the formazan product and the absorbance was measured at 540 nm in a Victor 3 V 1420 multi-label counter (Perkin Elmer, Akron, OH, USA).

### Identification of synergism and antagonism in drug combination studies

Drug concentrations used in combination studies were selected from the dose–response data for individual agents in 231C and TXT cells. Fraction affected (*Fa*) values were calculated as the percentage inhibition of cell viability, relative to DMSO control, as described by Chou [20]. Synergism, additivity, or antagonism in drug combinations was identified using the Chou–Talalay Combination Index (*CI*) method [20] and CompuSyn software (http://www.combosyn.com)

$$CI = \frac{{\left( D \right)_{1} }}{{(D_{x} )_{1} }} + \frac{{\left( D \right)_{2} }}{{(D_{x} )_{2} }},$$

where (*D*)_1_ and (*D*)_2_ represent the concentrations of Drug 1 and Drug 2 in the combination that produce an *Fa* value of *x*. (*D*_*x*_)_1_ and (*D*_*x*_)_2_ represent the concentrations of Drug 1 and Drug 2 that produce the same effect (*x*) when applied as single agents. *CI* values < 1, = 1, and > 1 indicate synergism, additivity, and antagonism, respectively.

Dose Reduction Index (*DRI*) values were also calculated to assess whether drug combinations could minimise the potential toxicity and adverse effects from higher doses of the individual drugs. *DRI* values indicate the calculated fold dose reductions that would achieve equivalent efficacy as the drugs used individually. (*DRI*)_1_ and (*DRI*)_2_ were determined for drugs 1 and 2

$$\left( {DRI} \right)_{1} = \frac{{\left( {D_{x} } \right)_{1} }}{{\left( D \right)_{1} }}$$

$$\left( {DRI} \right)_{2} = \frac{{\left( {D_{x} } \right)_{2} }}{{\left( D \right)_{2} }}.$$

Here, (*D*)_1_ and (*D*)_2_ represent the concentrations of Drug 1 and Drug 2 in the combination that produced an *Fa* of *x*. (*D*_*x*_)_1_ and (*D*_*x*_)_2_ represent the concentrations of Drug 1 and Drug 2 as single agents that would produce equivalent effects. *DRI* values > 1, < 1, and = 1 indicate favourable, unfavourable, and no dose reductions, respectively. *Fa-CI* and *Fa-DRI* plots were also constructed. In the *Fa-CI* plot, data points appearing below the line of additivity (*CI* = 1) indicate synergism, while those above the line indicate antagonism. In the *Fa-DRI* plot, data points appearing below the line of no dose reduction (*DRI* = 1) indicate a favourable dose reduction, while those above the line indicate an unfavourable dose reduction [20].

### Annexin V-FITC/7AAD staining

231C and TXT cells were seeded in duplicate on 12-well plates at a density of 8.0 × 10^4^ cells/well. Cells were treated with varying concentrations of ixabepilone and either gefitinib or vandetanib for 48 h (in DMSO, 0.1% final); control cells received an equivalent volume of solvent (DMSO) alone. Cells were then harvested by trypsinization, washed twice with cold PBS, and resuspended in binding buffer, the annexin V-FITC and 7AAD dyes were added, and flow cytometry was performed on a Gallios instrument using V1.2 software (Beckman Coulter).

### Western immunoblot analysis

MDA-MB 231 cells were seeded (8.5 × 10^5^ cells/100 mm plate) and, after 24 h, were treated with different concentrations of drugs alone and in combination (in DMSO, 0.1% final); control cells received an equivalent volume of solvent (DMSO) alone. Cells were harvested (~ 80–90% confluence) using trypsin/EDTA before lysis with Laemmli buffer (31.25 mM Tris–HCl, pH 6.8; 1% sodium dodecylsulfate; 12.5% glycerol; 0.005% bromophenol blue; 2.5% β-mercaptoethanol). Protein extracts [21] were electrophoresed on 12% sodium dodecylsulfate-polyacrylamide gels [22]. After transfer to nitrocellulose (Whatman, Dassel, Germany), the membranes were incubated with 5% non-fat dry milk in Tris-buffered saline containing Tween 20 (10 mM Tris; 100 mM NaCl; 0.1% Tween 20), washed in the same buffer, and incubated overnight at 4 °C with primary antibodies, essentially as described previously [23]. Detection was performed using IRDye conjugated goat anti-mouse or goat anti-rabbit IgG secondary antibody (1:10,000 dilution, 1 h, room temperature; Li-Cor Biosciences, Lincoln, NE) and analyzed with an Odyssey Infrared Imaging System (Li-Cor Biosciences). In preliminary analyses, densitometric signals were linearly related to protein loading.

### Statistical analysis

Data are expressed throughout as means ± SEM. All experiments were replicated on three occasions as indicated in figure legends. Data normality was confirmed using Studentized range testing [24]. Combination analysis was conducted as described by Chou [20]. Experimental data were analyzed by ANOVA and PLSD multiple comparison testing as indicated in figure legends (Statview, Abacus Corp, San Diego, CA or Excel, Microsoft Corp, Redmond, WA). Full statistical data, including F ratios, degrees of freedom, and *P *values, are available in Supplementary Materials.

## Results

### Activities of ixabepilone and other agents in parental and docetaxel-resistant MDA-MB-231 cell lines

**Fig. 1:**
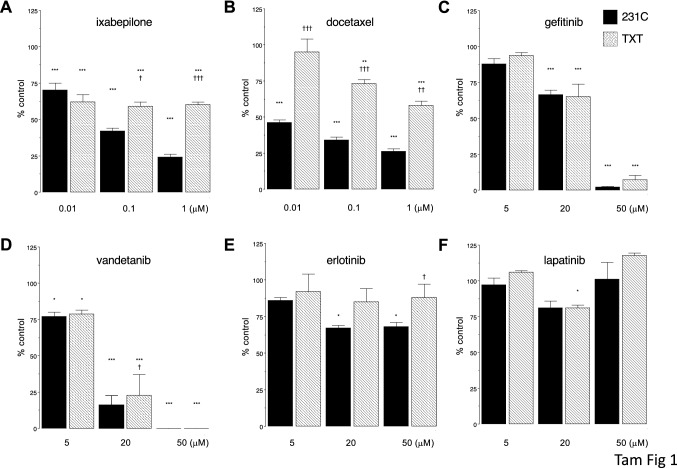
Inhibition of cell viability in parental MDA-MB-231 (231C; solid) and docetaxel-resistant MDA-MB-231 (TXT; hatching) cells by **A** ixabepilone, **B** docetaxel, **C** gefitinib, **D** vandetanib, **E** erlotinib, and **F** lapatinib. Different from corresponding control: *P < 0.05, ***P < 0.001. Different from corresponding findings in 231C cells: †*P* < 0.05, ††*P* < 0.01, †††*P* < 0.001. Data are mean ± SEM of *n* = 3 independent experiments and were analyzed by two-way ANOVA and PLSD testing

Ixabepilone effectively decreased the viability of parental 231C cells in a concentration-dependent manner (Fig. 1A). Ixabepilone was also active in docetaxel-resistant TXT cells, although not to the same extent as in 231C cells (Fig. 1A). In comparison, docetaxel was active in 231C cells but, as anticipated, its activity was much less pronounced in TXT cells (Fig. 1B). Thus, at a concentration of 1 µM, docetaxel decreased the viability of 231C and TXT cells to 26 ± 2% and 58 ± 3% of respective control.

Because the EGFR is a potentially promising drug target in TNBC, we tested four anti-EGFR TKIs—gefitinib, vandetanib, lapatinib, and erlotinib—for the capacity to decrease the viability of MDA-MB-231-derived cells. As shown in Fig. 1C, gefitinib (20 µM) decreased 231C and TXT cell viabilities to 67 ± 3% and 65 ± 9% of respective control (Fig. 1C). Vandetanib has been evaluated less extensively than gefitinib in TNBC. In the present study, the decreases in MTT reduction produced in 231C and TXT cells by vandetanib (20 µM) were to 16 ± 6% and 23 ± 14% of respective control. Lapatinib (Fig. 1E) and erlotinib (Fig. 1F) produced minimal inhibition of cell viability in both cell types even when tested at a concentration of 50 µM.

### The combination of ixabepilone and EGFR inhibitors decreases the viability of MDA-MB-231-derived cells

**Fig. 2:**
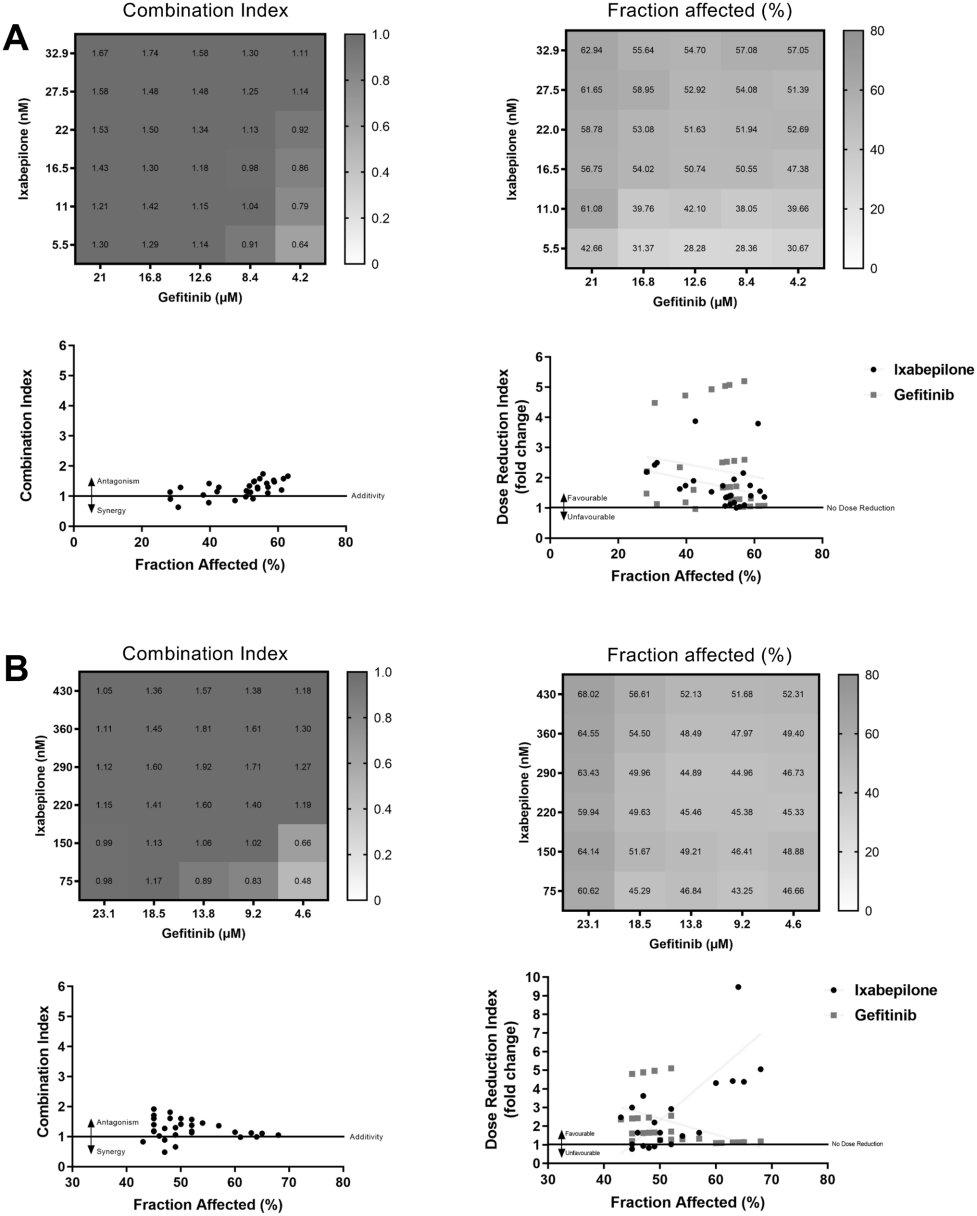
Analysis of combinations of ixabepilone and gefitinib on cell viability in **A** parental MDA-MB-231 (231C) and **B** docetaxel-resistant MDA-MB-231 (TXT) cells. In each panel: Upper left: Combination index values, Upper right: Fraction affected values (%), Lower left: Fraction affected (%) versus Combination index plot, and Lower right: Fraction affected (%) versus Dose Reduction Index plot

The activity of ixabepilone was assessed in combination with the EGFR inhibitors gefitinib and vandetanib. In 231C cells, most gefitinib and ixabepilone combinations (Suppl Table 1) produced low *Fa* levels and antagonism or moderate antagonism (*CI* range ~ 1–2)[20]. As shown in the *CI* plot (Fig. 2A), synergism (*CI* < 1) was observed at concentrations that produced low *Fa* values. For example, gefitinib (4.2 µM) and ixabepilone (16.5 nM) produced slight synergism (*CI* 0.86) but only a moderate *Fa* value of 47.38% (Fig. 2A). Despite these findings, the *DRI* values were relatively favourable for gefitinib and ixabepilone in the tested combinations (Fig. 2A). For example, gefitinib (21 µM) plus ixabepilone (22 nM) produced relatively small *DRI* values for gefitinib and ixabepilone of 1.05 and 1.74, respectively (Suppl Table 1).

Similarly, in TXT cells, gefitinib and ixabepilone combinations (Suppl Table 2) demonstrated antagonism (*CI* > 1). For example, gefitinib (23.1 µM) in combination with ixabepilone (290 nM) showed *CI* and *Fa* values of 1.12 and 63.43%, respectively (Fig. 2B); *DRI* values were again somewhat favourable for gefitinib and ixabepilone (Fig. 2B). Thus, for the combination of gefitinib (23.1 µM) and ixabepilone (290 nM), the *DRI* values were 1.12 and 4.42, respectively (Suppl Table 2).

**Fig. 3:**
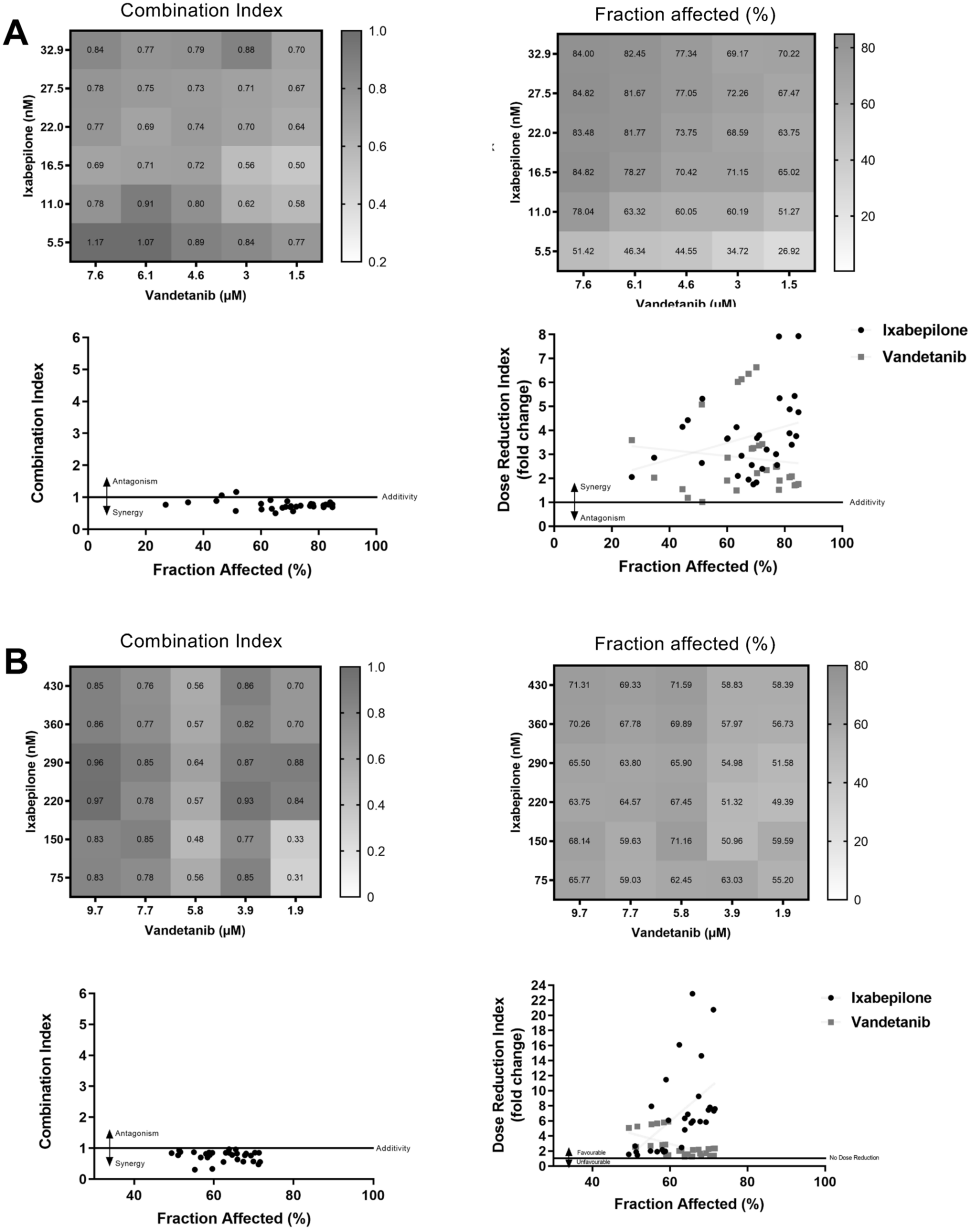
Analysis of combinations of ixabepilone and vandetanib on cell viability in **A** parental MDA-MB-231 (231C) and **B** docetaxel-resistant MDA-MB-231 (TXT) cells. In each panel: Upper left: Combination index values, Upper right: Fraction affected values (%), Lower left: Fraction affected (%) versus Combination index plot, and Lower right: Fraction affected (%) versus Dose Reduction Index plot

In contrast, most vandetanib and ixabepilone combinations demonstrated synergism and higher *Fa* values in 231C cells. Thus, vandetanib (1.5 µM) plus ixabepilone (16.5 nM) showed synergism (*CI* 0.50 and *Fa* 65%) (Fig. 3A). For this combination, very promising *DRI* values for vandetanib and ixabepilone were obtained − 6.14 and 2.95, respectively (Suppl Table 3). Similarly, most vandetanib and ixabepilone combinations also demonstrated synergism and high *Fa* values in TXT cells. Vandetanib (5.8 µM) in combination with ixabepilone (150 nM) showed synergism with *CI* and *Fa* values of 0.48 and 71.16%, respectively (Fig. 3B). In TXT cells, the *DRI* values for vandetanib and ixabepilone were 2.33 and 20.75, respectively (Suppl Table 4). Taken together, these data indicate that the combination of vandetanib and ixabepilone is synergistic and produced favourable dose reductions in both 231C and drug-resistant TXT cell lines. In contrast, the combination of gefitinib and ixabepilone produced antagonism, although small but favourable dose reductions were noted in both cell lines.

### Ixabepilone in combination with gefitinib and vandetanib modulates killing in MDA-MB-231-derived cells

**Fig. 4:**
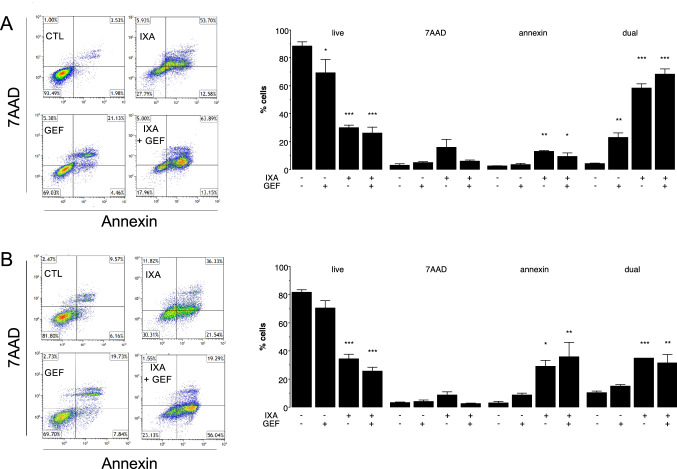
Analysis of annexin V/7AAD staining in **A** parental MDA-MB-231 (231C) and **B** docetaxel-resistant MDA-MB-231 (TXT) after treatment with the ixabepilone and gefitinib combination. In 231C cells: CTL, DMSO treatment; IXA, ixabepilone (22 nM); GEF, gefitinib (21 µM); IXA + GEF (ixabepilone 22 nM and gefitinib 21 µM). In TXT cells: CTL, DMSO treatment; IXA, ixabepilone (290 nM); GEF, gefitinib (23.1 µM); IXA + GEF (ixabepilone 290 nM and gefitinib 23.1 µM). Different from corresponding control: *P < 0.05, **P < 0.01, ***P < 0.001. Data are mean ± SEM of *n* = 3 independent experiments and were analyzed by one-way ANOVA and PLSD testing

In further studies, the impact of the combination of gefitinib and ixabepilone on cell death mechanisms was assessed in 231C cells using annexin V/7AAD staining. Ixabepilone (22 nM) as a single agent decreased the proportion of live cells from 88.3 ± 3.1% to 29.2 ± 2.0% (*P* < 0.001) and produced respective increases in early apoptotic (annexin V-stained) cells and late apoptotic/necrotic (dual-stained) cells to 5.3-fold (*P* < 0.01) and 13.8-fold (*P* < 0.001) of control (Fig. 4A). In agreement with combination studies, the inclusion of gefitinib (21 µM) produced no further increase in cell death over that produced by ixabepilone alone (Fig. 4A).

The combination of ixabepilone (290 nM) and gefitinib (23.1 µM) was also assessed in docetaxel-resistant TXT cells. Ixabepilone as a single agent decreased the proportion of live cells from 81.6 ± 2.0% to 34.2 ± 3.5% (*P* < 0.001; Fig. 4B), and increased early apoptosis and late apoptosis/necrosis in cells to 9.6-fold (*P* < 0.05) and 3.4-fold (*P* < 0.001) of respective control (Fig. 4B); the small increase in 7AAD staining did not attain statistical significance. Again, no further change in cell death was produced by the combination over that produced by ixabepilone alone (Fig. 4B).

**Fig. 5:**
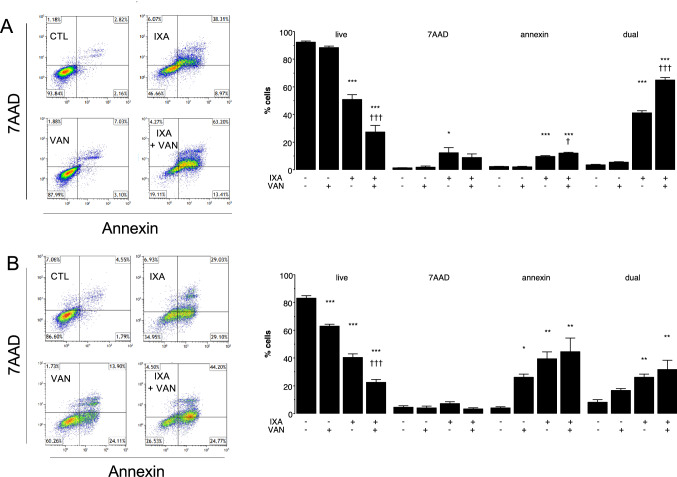
Analysis of annexin V/7AAD staining in **A** parental MDA-MB-231 (231C) and **B** docetaxel-resistant MDA-MB-231 (TXT) after treatment with the ixabepilone and vandetanib combination. In 231C cells: CTL, DMSO treatment; IXA, ixabepilone (16.5 nM); VAN, vandetanib (1.5 µM); IXA + VAN (ixabepilone 16.5 nM and vandetanib 1.5 µM). In TXT cells: CTL, DMSO treatment; IXA, ixabepilone (150 nM); VAN, vandetanib (5.8 µM); IXA + VAN (ixabepilone 150 nM and vandetanib 5.8 µM). Different from corresponding control: *P < 0.05, **P < 0.01, ***P < 0.001. Different from ixabepilone alone: †*P* < 0.05, †††*P* < 0.001. Data are mean ± SEM of *n* = 3 independent experiments and were analyzed by one-way ANOVA and PLSD testing

The impact of the vandetanib and ixabepilone combination on cell death pathways was also assessed using annexin V/7AAD staining. Ixabepilone (16.5 nM) as a single agent decreased the proportion of live cells from 92.2 ± 0.9% to 50.8 ± 3.5% control (*P* < 0.001), while there was increased staining by 7AAD (9.2-fold of control; *P* < 0.05), annexin V (4.1-fold of control; *P* < 0.001), and annexin V/7AAD (12.3-fold of control; *P* < 0.001; Fig. 5A). The combination of ixabepilone with vandetanib (1.5 µM) decreased the proportion of live cells to 27 ± 5%, and increased the proportions of early apoptotic (annexin V-stained) and late apoptosis/necrosis (dual-stained cells) to 12.0 ± 0.6% and 64.7 ± 2.1% of total, respectively (Fig. 5A).

The effect of the ixabepilone (150 nM)/vandetanib (5.8 µM) combination in TXT cells was also assessed. Ixabepilone (150 nM) as a single agent decreased the proportion of live cells from 83.1 ± 1.8% to 40.2 ± 2.7% (*P* < 0.001; Fig. 5B) and increased annexin V- (*P* < 0.01) and dual-stained cells (*P* < 0.01; Fig. 5B). The combination also markedly decreased the proportion of live cells compared with ixabepilone alone (*P* < 0.001; Fig. 5B). Together these findings indicate that the combination of ixabepilone with vandetanib, but not gefitinib, markedly increased killing in both 231C and TXT cells. The increased proportion of 231C over TXT cells that entered late-stage apoptosis in response to ixabepilone may have been due to drug resistance in TXT cells.

### The ixabepilone/vandetanib combination alters the expression of cleaved caspase-3 and Bcl-2 proteins in 231C and TXT cells

**Fig. 6:**
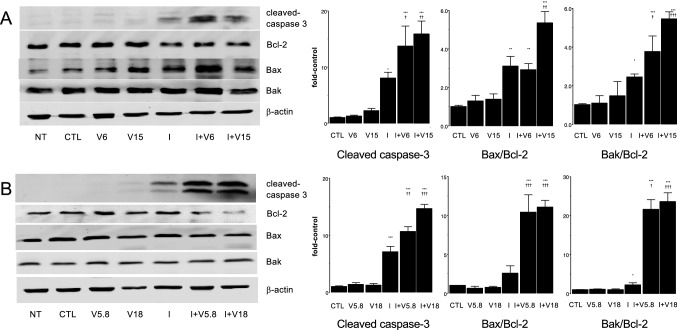
Western immunoblot analysis of cleaved caspase-3, Bcl-2, Bax, and Bak expression in 231C and TXT cells after treatment with the ixabepilone and vandetanib combination. **A** In 231C cells: CTL, DMSO treatment; I, ixabepilone (16.5 nM); V6, vandetanib (6 µM); V15, vandetanib (15 µM); I + V6 (ixabepilone 16.5 nM and vandetanib 6 µM); I + V15 (ixabepilone 16.5 nM and vandetanib 15 µM). **B** In TXT cells: CTL, DMSO treatment; I, ixabepilone (150 nM); V5.8, vandetanib (5.8 µM); V18, vandetanib (18 µM); I + V5.8 (ixabepilone 150 nM and vandetanib 5.8 µM); I + V18 (ixabepilone 150 nM and vandetanib 18 µM). Different from corresponding control: *P < 0.05, **P < 0.01, ***P < 0.001. Different from ixabepilone alone: †*P* < 0.05, ††*P* < 0.01, †††*P* < 0.001. Data are mean ± SEM of *n* = 3 independent experiments and were analyzed by one-way ANOVA and PLSD testing

To further assess the killing capacity of the ixabepilone/vandetanib combination, the expression of key apoptotic markers was evaluated by Western immunoblotting. In 231C cells, treatment with the combination of ixabepilone (16.5 nM) and vandetanib (6 and 15 μM; 24 h) increased cleaved caspase-3 expression to 13.7 ± 3.6- and 15.9 ± 2.3-fold of control, respectively (*P* < 0.001; Fig. 6A). In comparison, ixabepilone alone increased cleaved caspase-3 expression to 8.0 ± 1.0-fold of control, while vandetanib alone produced minor changes. We also assessed the effect of these treatments on the ratios of pro- (Bax and Bak) and anti-apoptotic Bcl-2 proteins (Bcl-2) as further indicators of apoptosis [25]. The ixabepilone/vandetanib combination markedly increased the Bax/Bcl-2 and Bak/Bcl-2 ratios in 231C cells.

Analogous studies were conducted in docetaxel-resistant TXT cells using ixabepilone (150 nM) and vandetanib (5.8 and 18 μM; 24 h). As shown in Fig. 6B, the combination again markedly increased cleaved caspase-3 expression over those produced by ixabepilone and vandetanib alone. Again, the Bax/Bcl-2 and Bak/Bcl-2 expression ratios were increased. Together, these findings indicate that the enhanced apoptosis that is produced in 231C and TXT cells by treatment with vandetanib in combination with ixabepilone is associated with enhanced caspase-3 cleavage and shifts in pro/anti-apoptotic protein ratios.

## Discussion

The principal finding that emerged from the present study is that the combination of the cytotoxic agent ixabepilone and the EGFR-targeted TKI vandetanib is synergistic in both parental 231C and docetaxel-resistant TXT cells. *DRI* values calculated from combination analysis suggested that several-fold dose reductions with ixabepilone/vandetanib might be achieved in the clinical setting. Synergism and effective *DRI*s were also produced by the combination in docetaxel-resistant TXT cells. Together, this suggests that the combination could now be assessed in TNBC patients, including those who may be resistant to first-line cytotoxic drugs like docetaxel.

The development of resistance to cytotoxic agents is a major problem in TNBC and results in extremely poor outcomes [26]. Overexpression of efflux transporters, such as ABCB1 and BCRP, is a major mechanism of resistance, although additional mechanisms have been reported. For example, tumors may exhibit decreased expression of solute carrier (SLC) transporters that are known to mediate the entry of drugs to cells [27, 28] or increased activity of CYP oxidases and other enzymes that play important roles in the biotransformation of drugs and other foreign compounds [29–35]. Such pharmacokinetic mechanisms decrease the active concentrations of anticancer agents within tumors, which decreases their anti-tumor activity. Additional resistance mechanisms may also be activated in tumors, including the dysregulation of intracellular signaling pathways that control tumor cell survival and apoptosis [35].

Ixabepilone is used to treat advanced TNBC after the emergence of resistance to first-line agents, such as the anthracyclines and taxanes [11]. Ixabepilone has certain advantages over these agents in that it is a substrate for the efflux transporter ABCB1, but not BCRP, which is also over-expressed in many tumors [12]. In consequence, the efflux of ixabepilone from tumor cells may be somewhat lower than for other cytotoxic drugs. Anti-cancer drug combinations are being used increasingly in the clinical treatment of cancer patients, because they are often more effective than single drug treatments. Combination analysis can be used in preclinical studies in cells to identify synergistic drug combinations that could be tested clinically [20]. This strategy was adopted in the present study.

Using drug combinations based on ixabepilone, the rational design of new drug strategies for use in TNBC could be improved if novel drug targets were identified. The EGFR is a viable target, at least in some TNBCs, because it is predictive of poor outcome and low rates of 5-year overall survival [9, 36, 37]. A number of clinical trials have been conducted in breast cancer using TKIs like gefitinib and erlotinib that target the EGFR. However, to date, outcomes with single agent anti-EGFR TKIs have been disappointing. For example, a Phase II trial of gefitinib (500 mg/day) produced no significant clinical benefit in patients with metastatic breast cancer who had been treated previously with taxanes or anthracyclines [38]. Interestingly, however, certain subpopulations of TNBC patients appeared to benefit from EGFR-targeted therapy. Thus, one phase II study reported that the combined use of the anti-EGFR antibody cetuximab and cisplatin produced an improved response over cisplatin alone in patients with metastatic TNBC [10]. In other studies, the combination of gefitinib and docetaxel produced a response rate of 54% in patients with metastatic breast cancer [39], and gefitinib in combination with epirubicin and cyclophosphamide improved the complete pathologic response in TNBC patients compared with non-TNBC patients [40].

The use of other cytotoxic drugs in combination with other EGFR TKIs could revive the clinical strategy. The activity of ixabepilone in drug-resistant TXT cells that was identified in the present study suggests that this drug has advantages over docetaxel and other cytotoxic agents in drug-resistant cells and could provide a firm basis for the development of new combinations for clinical evaluation. Interestingly, the use of the ixabepilone/capecitabine combination in patients with anthracycline- and taxane-resistant metastatic breast cancer was found to be promising [41]. Furthermore, in combination with gefitinib, ixabepilone was effective against TNBC-derived cancer stem cells in vitro and in tumor xenograft models in vivo [42]. In light of the antagonism exhibited by the ixabepilone–gefitinib combination in the present study, we assessed other EGFR TKIs.

Vandetanib is an EGFR TKI that has been approved for the treatment of patients with advanced medullary thyroid cancer [14]. Single agent vandetanib was found to induce tumor regression in vivo in TNBC patient-derived xenograft models with tumors that expressed high levels of EGFR [16] and showed clinical promise as an anti-proliferative agent in breast cancer in a small clinical trial (NCT01934335), although the sample size was very small. There have been few studies that have evaluated vandetanib in combination with cytotoxic agents. However, the vandetanib/docetaxel combination was effective in patients with previously treated non-small-cell lung cancer [15] and has also been evaluated in patients with advanced breast cancer. In the latter study, the combination produced a partial response in 14 patients (40%) and stable disease in another 11 (31%), while the corresponding numbers with docetaxel alone were 5 (17%) and 15 (52%) patients, respectively [43]. Even though these patient numbers were small, the findings from the present study suggest that further clinical evaluations of EGFR TKI/cytotoxic drug combinations, such as vandetanib/ixabepilone, in TNBC patients should be considered.

## Conclusions

In the present study, the combination of vandetanib and ixabepilone was synergistic in parental 231C cells and, from combination analysis, significant dose reductions for both drugs were predicted to be achievable in the clinical setting. There was also an increase in cell killing produced by the combination, as reflected by increased annexin V/7AAD staining and caspase-3 cleavage and increased expression ratios of pro-/anti-apoptotic Bcl-2 proteins. It is noteworthy that the ixabepilone/vandetanib was also synergistic in TXT cells and produced more effective killing than ixabepilone alone. The combination of ixabepilone with the clinically important EGFR TKI gefitinib was also active, but the cell killing produced by ixabepilone alone was not significantly enhanced by the TKI. Thus, the findings from the present study suggest that the ixabepilone/vandetanib combination could now be assessed clinically in patients with TNBC, including those in whom first-choice cytotoxic drugs have failed.

## Supplementary Information

Below is the link to the electronic supplementary material.

Supplementary file1 (PDF 3196 KB)

## Abbreviations

231C: MDA-MB-231 parental cell line
7AAD: 7-Aminoactinomycin D
ABCB1: ATP-binding cassette subfamily B member 1, or P-glycoprotein
BCRP: Breast cancer resistance protein
*CI*: Combination index
DMEM: Dulbecco's Modified Eagle's Medium
DMSO: Dimethylsulfoxide
*DRI*: Dose-reduction index
EGFR: Epidermal growth factor receptor
*Fa*: Fraction affected
FBS: Fetal bovine serum
PBS: Phosphate-buffered saline
SLC: Solute carrier
TKI: Tyrosine kinase inhibitor
TNBC: Triple-negative breast cancer
TXT: Docetaxel-resistant MDA-MB-231 cells

## Declarations

### Data availability

All data generated or analyzed during this study are included in the article and its supplementary information files.
