# Supplementary material for: The ixabepilone and vandetanib combination shows synergistic activity in docetaxel-resistant MDA-MB-231 breast cancer cells
Source: Pharmacol Rep. 2022 Jul 30;74(5):998–1010. doi: 10.1007/s43440-022-00396-7 (PMC9584993; doi:10.1007/s43440-022-00396-7)
Supplement: Supplementary file 1 — Supplementary file1 (PDF 3196 KB) [file 43440_2022_396_MOESM1_ESM.pdf]

The ixabepilone and vandetanib combination shows synergistic activity in docetaxel-resistant MDA-MB-231 breast cancer cells

Stanton Tam,[a,1] Yassir Al-Zubaidi,[a,‡,1] Md Khalilur Rahman,[a,b] Kirsi Bourget,[a,b] Fanfan Zhou[b] and Michael Murray[a,b,*]

Pharmacogenomics and Drug Development Group, [a]Discipline of Pharmacology, School of Medical Sciences, and [b]Sydney Pharmacy School, Faculty of Medicine and Health, University of Sydney, New South Wales, 2006, Australia,

[1]These authors contributed equally.

[‡]Present address: College of Pharmacy, The University of Mashreq, Baghdad, Iraq.

[*]To whom correspondence should be addressed: michael.murray@sydney.edu.au, Sydney Pharmacy School, Faculty of Medicine and Health, University of Sydney, NSW 2006, Australia

SUPPLEMENTARY MATERIAL **pages**

Supplementary Tables	data used to derive Figs 2 and 3	3-6
Supplementary Analysis	original data and statistical analysis in Figs 1, 4, 5 and 6	7-35
Supplementary Figures 1-10	full Westerns for the data shown in Fig 6	36-45

Supplementary Table 1: Dose Reduction Index (DRI) and Fraction affected (Fa) values for ixabepilone and gefitinib combinations in parental MDA-MB-231 (231C) cells.

**Concentration of drug in combination**		**Fraction Affected (%)**	**Concentration of drug alone to achieve same Fa**		**Dose Reduction Index, DRI (fold decrease)**	
Ixabepilone (nM)	*Gefitinib (µM)*		*Ixabepilone (nM)*	*Gefitinib (µM)*	*Ixabepilone*	*Gefitinib*
5.5	21.0	42.66	21.3	20.2	3.87	0.96
5.5	16.8	31.37	13.7	18.9	2.50	1.13
5.5	12.6	28.28	12	18.5	2.19	1.47
5.5	8.4	28.36	12.1	18.6	2.19	2.21
5.5	4.2	30.67	13.3	18.8	2.43	4.48
11.0	21.0	61.08	41.7	22.4	3.79	1.06
11.0	16.8	39.76	19.1	19.9	1.74	1.18
11.0	12.6	42.10	20.9	20.1	1.90	1.60
11.0	8.4	38.05	17.9	19.7	1.63	2.34
11.0	4.2	39.66	19.1	19.9	1.73	4.72
16.5	21.0	56.75	35.5	21.8	2.15	1.04
16.5	16.8	54.02	32.1	21.5	1.95	1.28
16.5	12.6	50.74	28.6	21.1	1.73	1.67
16.5	8.4	50.55	28.4	21.1	1.72	2.51
16.5	4.2	47.38	25.3	20.7	1.53	4.93
22.0	21.0	58.78	38.3	22.1	1.74	1.05
22.0	16.8	53.08	31.1	21.4	1.41	1.27
22.0	12.6	51.63	29.5	21.2	1.34	1.68
22.0	8.4	51.94	29.8	21.3	1.36	2.53
22.0	4.2	52.69	30.6	21.3	1.39	5.07
27.5	21.0	61.65	42.6	22.4	1.55	1.07
27.5	16.8	58.95	38.5	22.1	1.40	1.31
27.5	12.6	52.92	30.9	21.4	1.12	1.69
27.5	8.4	54.08	32.2	21.5	1.17	2.56
27.5	4.2	51.39	29.2	21.2	1.06	5.04
32.9	21.0	62.94	44.8	22.6	1.36	1.07
32.9	16.8	55.64	34.1	21.7	1.04	1.29
32.9	12.6	54.70	32.9	21.6	1.00	1.71
32.9	8.4	57.08	35.9	21.9	1.09	2.60
32.9	4.2	57.05	35.9	21.9	1.09	5.20

Supplementary Table 2: Dose Reduction Index (DRI) and Fraction affected (Fa) values for ixabepilone and gefitinib combinations in docetaxel-resistant MDA-MB-231 (TXT) cells.

**Concentration of drug in combination**		**Fraction Affected (%)**	**Concentration of drug alone to achieve same Fa**		**Dose Reduction Index, DRI (fold decrease)**	
Ixabepilone (nM)	*Gefitinib (µM)*		*Ixabepilone (nM)*	*Gefitinib (µM)*	*Ixabepilone*	*Gefitinib*
75	23.1	60.62	1048	25.4	13.97	1.10
75	18.5	45.29	225	22.1	2.99	1.20
75	13.8	46.84	272	22.5	3.62	1.63
75	9.2	43.25	185	21.7	2.47	2.36
75	4.6	46.66	272	22.5	3.62	4.89
150	23.1	64.14	1421	26.1	9.47	1.13
150	18.5	51.67	438	23.5	2.92	1.27
150	13.8	49.21	329	22.9	2.19	1.66
150	9.2	46.41	247	22.3	1.65	2.42
150	4.6	48.88	329	22.9	2.19	4.97
220	23.1	59.94	949	25.2	4.31	1.09
220	18.5	49.63	362	23.1	1.64	1.25
220	13.8	45.46	225	22.1	1.02	1.60
220	9.2	45.38	225	22.1	1.02	2.40
220	4.6	45.33	225	22.1	1.02	4.81
290	23.1	63.43	1282	25.9	4.42	1.12
290	18.5	49.96	362	23.1	1.25	1.25
290	13.8	44.89	225	22.1	0.77	1.60
290	9.2	44.96	225	22.1	0.77	2.40
290	4.6	46.73	272	22.5	0.94	4.89
360	23.1	64.55	1576	26.3	4.38	1.14
360	18.5	54.5	530	23.9	1.47	1.29
360	13.8	48.49	299	22.7	0.83	1.64
360	9.2	47.97	299	22.7	0.83	2.47
360	4.6	49.4	329	22.9	0.91	4.97
430	23.1	68.02	2172	27.1	5.05	1.17
430	18.5	56.61	707	24.5	1.64	1.33
430	13.8	52.13	438	23.5	1.02	1.70
430	9.2	51.68	438	23.5	1.02	2.55
430	4.6	52.31	438	23.5	1.02	5.10

Supplementary Table 3: Dose Reduction Index (DRI) and Fraction affected (Fa) values for ixabepilone and vandetanib combinations in parental MDA-MB-231 (231C) cells.

**Concentration of drug in combination**		**Fraction Affected, Fa (%)**	**Concentration of drug alone to achieve same Fa**		**Dose Reduction Index, DRI (fold decrease)**	
Ixabepilone (nM)	*Vandetanib (µM)*		*Ixabepilone (nM)*	*Vandetanib (µM)*	*Ixabepilone*	*Vandetanib*
5.5	7.6	51.42	29.3	7.8	5.32	1.02
5.5	6.1	46.34	24.4	7.3	4.43	1.19
5.5	4.6	44.55	22.8	7.1	4.15	1.55
5.5	3.0	34.72	15.7	6.2	2.86	2.03
5.5	1.5	26.92	11.3	5.5	2.06	3.60
11.0	7.6	78.04	87.1	11.6	7.92	1.52
11.0	6.1	63.32	45.5	9.1	4.13	1.50
11.0	4.6	60.05	40.1	8.7	3.65	1.91
11.0	3.0	60.19	40.3	8.7	3.67	2.87
11.0	1.5	51.27	29.1	7.7	2.65	5.09
16.5	7.6	84.82	131	13.4	7.94	1.76
16.5	6.1	78.27	88.2	11.6	5.35	1.91
16.5	4.6	70.42	60.7	10.1	3.68	2.22
16.5	3.0	71.15	62.7	10.3	3.80	3.37
16.5	1.5	65.02	48.6	9.3	2.95	6.14
22.0	7.6	83.48	119.6	13.0	5.44	1.71
22.0	6.1	81.77	107.4	12.5	4.88	2.05
22.0	4.6	73.75	70.5	10.7	3.21	2.34
22.0	3.0	68.59	56.2	9.8	2.55	3.24
22.0	1.5	63.75	46.2	9.2	2.10	6.02
27.5	7.6	84.82	131	13.4	4.76	1.77
27.5	6.1	81.67	106.8	12.5	3.88	2.05
27.5	4.6	77.05	82.8	11.4	3.01	2.49
27.5	3.0	72.26	65.9	10.4	2.40	3.43
27.5	1.5	67.47	53.6	9.7	1.95	6.36
32.9	7.6	84.00	123.8	13.2	3.76	1.73
32.9	6.1	82.45	112.1	12.7	3.41	2.08
32.9	4.6	77.34	84	11.4	2.55	2.50
32.9	3.0	69.17	57.6	9.9	1.75	3.26
32.9	1.5	70.22	60.2	10.1	1.83	6.64

Supplementary Table 4: Dose Reduction Index (DRI) and Fraction affected (Fa) values for ixabepilone and vandetanib combinations in docetaxel-resistant MDA-MB-231 (TXT) cells.

Concentration of drug in combination		Fraction Affected (%)	Concentration of drug alone to achieve same Fa		Dose Reduction Index, DRI (fold decrease)	
Ixabepilone (nM)	*Vandetanib (µM)*		*Ixabepilone (nM)*	*Vandetanib (µM)*	*Ixabepilone*	*Vandetanib*
75	9.7	65.77	1715	12.3	22.86	1.27
75	7.7	59.03	860	11.1	11.46	1.44
75	5.8	62.45	1207	11.7	16.09	2.02
75	3.9	63.03	185	8.8	2.47	2.25
75	1.9	55.2	594	10.5	7.92	5.52
150	9.7	68.14	2196	12.8	14.64	1.32
150	7.7	59.63	912	11.2	6.08	1.46
150	5.8	71.16	3113	13.5	20.75	2.33
150	3.9	50.96	398	9.9	2.65	2.53
150	1.9	59.59	912	11.2	6.08	5.90
220	9.7	63.75	1392	12.0	6.33	1.23
220	7.7	64.57	1512	12.1	6.87	1.57
220	5.8	67.45	2035	12.7	9.25	2.19
220	3.9	51.32	409	9.9	1.86	2.54
220	1.9	49.39	342	9.6	1.55	5.08
290	9.7	65.5	1661	12.3	5.73	1.27
290	7.7	63.8	1392	12.0	4.80	1.55
290	5.8	65.9	1733	12.4	5.98	2.13
290	3.9	54.98	583	10.5	2.01	2.68
290	1.9	51.58	421	10.0	1.45	5.24
360	9.7	70.26	2807	13.3	7.80	1.37
360	7.7	67.78	2125	12.8	5.90	1.66
360	5.8	69.89	2682	13.2	7.45	2.28
360	3.9	57.97	779	10.9	2.17	2.81
360	1.9	56.73	687	10.7	1.91	5.65
430	9.7	71.31	3149	13.6	7.32	1.40
430	7.7	69.33	2507	13.1	5.83	1.70
430	5.8	71.59	3261	13.6	7.58	2.35
430	3.9	58.83	843	11.1	1.96	2.84
430	1.9	58.39	810	11.0	1.89	5.79

Supplementary analysis 1: Original data in Figure 1 and Two-Factor Anova With Replication

Ixabepilone (ixa), docetaxel (doc), gefitinib (gef), vandetanib (vand), erlotinib (erl) and lapatinib (lap) were tested in docetaxel-sensitive 231C and docetaxel-resistant TXT cells for the capacity to inhibit cell viability (MTT reduction; % control values). Drugs were tested at the low, medium and high concentrations that are indicated in Figure 1.

	control	ixa low	ixa med	ixa high	doc low	doc mid	doc high	gef low	gef mid	gef high
231C	101	69	42	23	46.5	34	22.5	87.6	60.6	1.8
	110.5	79	38.5	31	41.5	30.5	26	81.2	87.8	2.9
	88	62	45.5	28.5	49.2	37.5	29.5	93.4	81.6	1.3
TXT	98	62.6	53.8	60	110.5	74	56	93	65	5.3
	90.5	53	64.2	63	79.5	78	54	98	80	12.5
	111.5	70.4	59	57	95	67.5	64	89	50	4.1

	vand low	vand mid	vand high	erl low	erl mid	erl high	lap low	lap mid	lap high
231C	77	3.1	0	86.5	67.7	68	100.2	81.5	121.8

	71.6	3.1	0	89	70.8	73.2	104.1	88.7	101
	82.4	2.8	0	82	62.5	62.8	86.9	72.8	80.2
TXT	78	48.3	0	92	69.5	90	107.7	81	121.5
	85	10	0	71.2	85	111.5	104.3	84.5	118
	74	20	0	112.8	100.5	72.5	106	77.5	114.6

ANOVA

Source of Variation	*SS*	*df*	*MS*	*F*	*P-value*	*F crit*
Cell type	5346.42535	1	5346.42535	58.5668097	5.0707E-11	3.96675978
treatments	109796.329	18	6099.79608	66.8195239	4.19127E-39	1.74118919
Interaction	7265.47965	18	403.637758	4.42160401	2.26194E-06	1.74118919
Within	6937.86	76	91.2876316			
Total	129346.094	113				

Three null hypotheses (that the means of observations grouped by cell type are the same; that the means of observations grouped by treatmentare the same; and that there is no interaction between the two factors) were all rejected because the F values exceeded the critical values.

Differences between mean values of groups:

231C cells

Comparison	mean difference	critical difference	p value
Control vs ixabepilone low	30.0	12.44	0.0001
Control vs ixabepilone mid	58.0		<0.0001
Control vs ixabepilone high	72.7		<0.0001
Control vs docetaxel low	54.0	14.05	<0.0001
Control vs docetaxel mid	66.0		<0.0001
Control vs docetaxel high	74.0		<0.0001
Control vs gefitinib low	12.2	14.20	0.0873
Control vs gefitinib mid	33.7		0.0001
Control vs gefitinib high	98.0		<0.0001
Control vs vandetanib low	23.0	15.53	0.0063
Control vs vandetanib mid	97.0		<0.0001
Control vs vandetanib high	100.0		<0.0001
Control vs erlotinib low	14.0	22.51	0.2059
Control vs erlotinib mid	33.0		0.0068
Control vs erlotinib high	32.0		0.0082

Control vs lapatinib low	3.0	17.43	0.7200
Control vs lapatinib mid	19.0		0.0345
Control vs lapatinib low	1.0		0.9047

TXT cells

Comparison	mean difference	critical difference	p value
Control vs ixabepilone low	38.0	12.44	<0.0001
Control vs ixabepilone mid	41.0		<0.0001
Control vs ixabepilone high	40.0		<0.0001
Control vs docetaxel low	5.0	14.05	0.4615
Control vs docetaxel mid	27.0		0.0009
Control vs docetaxel high	42.0		<0.0001
Control vs gefitinib low	6.5	14.20	0.3463
Control vs gefitinib mid	35.0		<0.0001
Control vs gefitinib high	92.7		<0.0001
Control vs vandetanib low	21.0	15.53	0.0112
Control vs vandetanib mid	73.9		<0.0001
Control vs vandetanib high	100.0		<0.0001
Control vs erlotinib low	8.0	22.51	0.4621

Control vs erlotinib mid	15.0		0.1769
Control vs erlotinib high	8.7		0.4264
Control vs lapatinib low	25.0	17.43	0.0078
Control vs lapatinib mid	12.0		0.1632
Control vs lapatinib low	37.0		0.0004

Supplementary analysis for Figure 4A: Original data and One way ANOVA with replication

Ixabepilone and gefitinib were tested in docetaxel-sensitive 231C cells for the capacity to inhibit activate cell killing (annexin V/7AAD staining; values are cell proportions as %). Drugs were tested at the concentrations that are indicated in Figure 4A.

Treatment	live	7AAD	annexin	dual
control	82.8	5.0	2.9	5.1
	88.5	3.1	2.4	4.1
	93.5	1.0	2.0	3.5
gefitinib	69.0	5.4	4.5	21.1
	86.0	5.8	1.2	29.4
	52.2	3.3	4.2	17.6
ixabepilone	34.0	15.1	11.5	57.1
	28.0	26.1	14.3	64.0
	27.8	5.9	12.6	53.7
Ixabepilone + gefitinib	26.1	5.0	4.4	64.8
	33.6	7.7	9.9	75.8
	18.0	5.0	13.2	63.9

Live cells

ANOVA Table for LIVE

	DF	Sum of Squares	Mean Square	F-Value	P-Value
Column 1	3	8298.573	2766.191	28.608	.0001
Residual	8	773.547	96.693		

Model II estimate of between component variance: 889.833

Means Table for LIVE
Effect: Column 1

	Count	Mean	Std. Dev.	Std. Err.
CONTROL	3	88.267	5.354	3.091
GEFITINIB	3	69.067	16.900	9.757
IXABEPILONE	3	29.933	3.523	2.034
IXABEPILONE + GEFITINIB	3	25.933	7.751	4.475

Comparison	mean difference	critical difference	p value
Control vs gefitinib	19.2	18.52	0.0438
Control vs ixabepilone	58.3		<0.0001
Control vs gefitinib plus ixabepilone	62.3		<0.0001

7AAD-stained cells

ANOVA Table for 7AAD

	DF	Sum of Squares	Mean Square	F-Value	P-Value
Column 1	3	290.373	96.791	3.503	.0694
Residual	8	221.033	27.629		

Model II estimate of between component variance: 23.054

Means Table for 7AAD
Effect: Column 1

	Count	Mean	Std. Dev.	Std. Err.
CONTROL	3	3.033	2.001	1.155
GEFITINIB	3	4.833	1.343	.775
IXABEPILONE	3	15.700	10.113	5.839
IXABEPILONE + GEFITINIB	3	5.900	1.559	.900

P value not significant: no further analysis

Annexin V-stained cells

ANOVA Table for annexin

	DF	Sum of Squares	Mean Square	F-Value	P-Value
Column 1	3	218.569	72.856	11.525	.0028
Residual	8	50.573	6.322		

Model II estimate of between component variance: 22.178

Means Table for annexin
Effect: Column 1

	Count	Mean	Std. Dev.	Std. Err.
CONTROL	3	2.433	.451	.260
GEFITINIB	3	3.300	1.825	1.054
IXABEPILONE	3	12.800	1.411	.814
IXABEPILONE + GEFITINIB	3	9.167	4.446	2.567

Comparison	mean difference	critical difference	p value
Control vs gefitinib	0.9	4.73	0.6840
Control vs ixabepilone	10.4		0.0010
Control vs gefitinib plus ixabepilone	6.73		0.0112

Dual Annexin V/7AAD-stained cells

ANOVA Table for dual

	DF	Sum of Squares	Mean Square	F-Value	P-Value
Column 1	3	8083.729	2694.576	99.038	<.0001
Residual	8	217.660	27.207		

Model II estimate of between component variance: 889.123

Means Table for dual
Effect: Column 1

	Count	Mean	Std. Dev.	Std. Err.
CONTROL	3	4.233	.808	.467
GEFITINIB	3	22.700	6.061	3.499
IXABEPILONE	3	58.267	5.248	3.030
IXABEPILONE + GEFITINIB	3	68.167	6.626	3.825

Comparison	mean difference	critical difference	p value
Control vs gefitinib	18.5	9.8	0.0025
Control vs ixabepilone	54.0		<0.0001
Control vs gefitinib plus ixabepilone	63.9		<0.0001

Supplementary analysis for Figure 4B: Original data and One way ANOVA with replication

Ixabepilone and gefitinib were tested in docetaxel-resistant TXT cells for the capacity to inhibit activate cell killing (annexin V/7AAD staining; values are cell proportions as %). Drugs were tested at the concentrations that are indicated in Figure 4B.

Treatment	live	7AAD	annexin	dual
control	81.8	2.5	6.2	9.6
	85.0	4.2	5.0	12.3
	78.1	2.7	1.0	7.9
gefitinib	76.8	6.0	8.6	14.7
	69.7	2.7	7.8	19.7
	71.3	4.2	6.1	12.6
ixabepilone	30.3	11.8	21.5	36.3
	41.6	9.5	37.9	34.1
	30.8	4.1	26.9	34.1
Ixabepilone + gefitinib	31.0	3.6	29.8	40.0
	23.1	1.6	56.0	19.3
	22.8	2.1	21.6	34.6

Live cells

ANOVA Table for live

	DF	Sum of Squares	Mean Square	F-Value	P-Value
Column 1	3	6678.222	2226.074	57.943	<.0001
Residual	8	307.347	38.418		

Model II estimate of between component variance: 729.219

Means Table for live
Effect: Column 1

	Count	Mean	Std. Dev.	Std. Err.
CONTROL	3	81.633	3.453	1.994
GEFITINIB	3	70.467	9.088	5.247
IXABEPILONE	3	34.233	6.127	3.538
IXABEPILONE + GEFITINIB	3	25.633	4.650	2.685

Comparison	mean difference	critical difference	p value
Control vs gefitinib	11.2	11.7	0.0584
Control vs ixabepilone	47.4		<0.0001
Control vs gefitinib plus ixabepilone	56.0		<0.0001

7AAD-stained cells

ANOVA Table for 7AAD

	DF	Sum of Squares	Mean Square	F-Value	P-Value
Column 1	3	66.473	22.158	3.722	.0609
Residual	8	47.627	5.953		

Model II estimate of between component variance: 5.401

Means Table for 7AAD
Effect: Column 1

	Count	Mean	Std. Dev.	Std. Err.
CONTROL	3	3.133	.929	.536
GEFITINIB	3	3.967	2.159	1.247
IXABEPILONE	3	8.467	4.148	2.395
IXABEPILONE + GEFITINIB	3	2.433	1.041	.601

P value not significant: no further analysis

Annexin V-stained cells

ANOVA Table for annexin

	DF	Sum of Squares	Mean Square	F-Value	P-Value
Column 1	3	2229.243	743.081	7.555	.0101
Residual	8	786.847	98.356		

Model II estimate of between component variance: 214.908

Means Table for annexin
Effect: Column 1

	Count	Mean	Std. Dev.	Std. Err.
CONTROL	3	3.000	2.000	1.155
GEFITINIB	3	8.600	2.500	1.443
IXABEPILONE	3	28.833	7.767	4.485
IXABEPILONE + GEFITINIB	3	35.800	17.968	10.374

Comparison	mean difference	critical difference	p value
Control vs gefitinib	5.6	18.7	0.5088
Control vs ixabepilone	25.8		0.0128
Control vs gefitinib plus ixabepilone	32.8		0.0037

Dual Annexin V/7AAD-stained cells

ANOVA Table for dual

	DF	Sum of Squares	Mean Square	F-Value	P-Value
Column 1	3	1312.600	437.533	14.046	.0015
Residual	8	249.207	31.151		

Model II estimate of between component variance: 135.461

Means Table for dual
Effect: Column 1

	Count	Mean	Std. Dev.	Std. Err.
CONTROL	3	10.100	2.200	1.270
GEFITINIB	3	14.700	2.100	1.212
IXABEPILONE	3	34.567	.252	.145
IXABEPILONE + GEFITINIB	3	31.300	10.737	6.199

Comparison	mean difference	critical difference	p value
Control vs gefitinib	4.6	10.5	0.3423
Control vs ixabepilone	24.5		0.0007
Control vs gefitinib plus ixabepilone	21.2		0.0016

Supplementary analysis for Figure 5A: Original data and One way ANOVA with replication

Ixabepilone and vandetanib were tested in docetaxel-sensitive 231C cells for the capacity to inhibit activate cell killing (annexin V/7AAD staining; values are cell proportions as %). Drugs were tested at the concentrations that are indicated in Figure 5A.

Treatment	live	7AAD	annexin	dual
control	92.0	1.5	2.7	4.8
	93.8	1.2	2.2	2.8
	90.7	1.3	2.1	2.4
vandetanib	90.4	3.3	0.9	5.1
	88.0	1.9	3.1	7.0
	86.9	0.3	1.9	4.5
ixabepilone	49.1	12.0	9.0	44.4
	57.5	18.8	10.9	39.4
	46.7	6.1	9.0	38.3
Ixabepilone + vandetanib	19.1	4.3	13.4	63.2
	27.3	8.9	11.7	61.5
	35.4	13.2	11.2	68.6

Live cells

ANOVA Table for live

	DF	Sum of Squares	Mean Square	F-Value	P-Value
Column 1	3	8778.656	2926.219	103.433	<.0001
Residual	8	226.327	28.291		

Model II estimate of between component variance: 965.976

Means Table for live
Effect: Column 1

	Count	Mean	Std. Dev.	Std. Err.
control	3	92.167	1.557	.899
vandetanib	3	88.433	1.845	1.065
ixa	3	50.833	5.991	3.459
ixa + van	3	27.067	8.452	4.880

Comparison	mean difference	critical difference	p value
Control vs vandetanib	3.7	10.0	0.4150
Control vs ixabepilone	41.3		<0.0001
Control vs vandetanib plus ixabepilone	65.1		<0.0001

7AAD-stained cells

ANOVA Table for 7AAD

	DF	Sum of Squares	Mean Square	F-Value	P-Value
Column 1	3	257.636	85.879	5.374	.0255
Residual	8	127.833	15.979		

Model II estimate of between component variance: 23.3

Means Table for 7AAD
Effect: Column 1

	Count	Mean	Std. Dev.	Std. Err.
control	3	1.333	.153	.088
vandetanib	3	1.800	1.500	.866
ixa	3	12.200	6.502	3.754
ixa + van	3	8.833	4.400	2.541

Comparison	mean difference	critical difference	p value
Control vs vandetanib	0.5	7.5	0.8898
Control vs ixabepilone	10.9		0.0104
Control vs vandetanib plus ixabepilone	7.5		0.0506

Annexin V-stained cells

ANOVA Table for annexin

	DF	Sum of Squares	Mean Square	F-Value	P-Value
Column 1	3	234.082	78.027	84.051	<.0001
Residual	8	7.427	.928		

Model II estimate of between component variance: 25.7

Means Table for annexin
Effect: Column 1

	Count	Mean	Std. Dev.	Std. Err.
control	3	2.333	.321	.186
vandetanib	3	1.933	1.050	.606
ixa	3	9.533	1.193	.689
ixa + van	3	12.033	1.041	.601

Comparison	mean difference	critical difference	p value
Control vs vandetanib	0.4	1.8	0.6249
Control vs ixabepilone	7.2		<0.0001
Control vs vandetanib plus ixabepilone	9.7		<0.0001

Dual Annexin V/7AAD-stained cells

ANOVA Table for dual

	DF	Sum of Squares	Mean Square	F-Value	P-Value
Column 1	3	7920.103	2640.034	447.843	<.0001
Residual	8	47.160	5.895		

Model II estimate of between component variance: 878.046

Means Table for dual
Effect: Column 1

	Count	Mean	Std. Dev.	Std. Err.
control	3	3.333	1.286	.742
vandetanib	3	5.333	.971	.561
ixa	3	41.133	2.831	1.634
ixa + van	3	64.700	3.601	2.079

Comparison	mean difference	critical difference	p value
Control vs vandetanib	2.0	4.6	0.3426
Control vs ixabepilone	37.8		<0.0001
Control vs vandetanib plus ixabepilone	61.4		<0.0001

Supplementary analysis for Figure 5B: Original data and One way ANOVA with replication

Ixabepilone and vandetanib were tested in docetaxel-resistant TXT cells for the capacity to inhibit activate cell killing (annexin V/7AAD staining; values are cell proportions as %). Drugs were tested at the concentrations that are indicated in Figure 5B.

Treatment	live	7AAD	annexin	dual
control	82.0	2.7	5.6	9.0
	86.6	7.1	1.8	4.6
	80.7	3.2	4.0	10.6
vandetanib	65.3	6.1	30.9	19.2
	62.9	4.1	23.1	16.4
	60.3	1.7	24.1	13.9
ixabepilone	42.0	4.3	43.0	21.0
	43.7	9.9	45.8	27.7
	35.0	6.9	29.1	29.0
Ixabepilone + vandetanib	21.0	3.9	56.3	29.0
	26.5	4.5	24.8	44.2
	19.5	0.8	52.5	21.5

Live cells

ANOVA Table for live

	DF	Sum of Squares	Mean Square	F-Value	P-Value
Column 1	3	6309.223	2103.074	165.890	<.0001
Residual	8	101.420	12.677		

Model II estimate of between component variance: 696.799

Means Table for live
Effect: Column 1

	Count	Mean	Std. Dev.	Std. Err.
control	3	83.100	3.100	1.790
vand	3	62.833	2.501	1.444
ixa	3	40.233	4.611	2.662
vand + ixa	3	22.333	3.686	2.128

Comparison	mean difference	critical difference	p value
Control vs vandetanib	20.3	6.7	0.0001
Control vs ixabepilone	42.9		<0.0001
Control vs vandetanib plus ixabepilone	60.8		<0.0001

7AAD-stained cells

ANOVA Table for 7AAD

	DF	Sum of Squares	Mean Square	F-Value	P-Value
Column 1	3	26.233	8.744	1.558	.2735
Residual	8	44.907	5.613		

Model II estimate of between component variance: 1.044

Means Table for 7AAD
Effect: Column 1

	Count	Mean	Std. Dev.	Std. Err.
control	3	4.333	2.409	1.391
vand	3	3.967	2.203	1.272
ixa	3	7.033	2.802	1.618
vand + ixa	3	3.067	1.986	1.146

P value not significant: no further analysis

Annexin V-stained cells

ANOVA Table for annexin

	DF	Sum of Squares	Mean Square	F-Value	P-Value
Column 1	3	2969.563	989.854	9.966	.0045
Residual	8	794.613	99.327		

Model II estimate of between component variance: 296.843

Means Table for annexin
Effect: Column 1

	Count	Mean	Std. Dev.	Std. Err.
control	3	3.800	1.908	1.102
vand	3	26.033	4.244	2.450
ixa	3	39.300	8.944	5.164
vand + ixa	3	44.533	17.195	9.927

Comparison	mean difference	critical difference	p value
Control vs vandetanib	22.2	18.8	0.0258
Control vs ixabepilone	35.5		0.0024
Control vs vandetanib plus ixabepilone	40.7		0.0010

Dual Annexin V/7AAD-stained cells

ANOVA Table for dual

	DF	Sum of Squares	Mean Square	F-Value	P-Value
Column 1	3	966.656	322.219	7.632	.0098
Residual	8	337.753	42.219		

Model II estimate of between component variance: 93.333

Means Table for dual
Effect: Column 1

	Count	Mean	Std. Dev.	Std. Err.
control	3	8.067	3.107	1.794
vand	3	16.500	2.651	1.531
ixa	3	25.900	4.293	2.479
vand + ixa	3	31.567	11.566	6.677

Comparison	mean difference	critical difference	p value
Control vs vandetanib	8.4	12.2	0.1506
Control vs ixabepilone	17.8		0.0099
Control vs vandetanib plus ixabepilone	23.5		0.0022

Supplementary analysis for Figure 6A: Original data and One way ANOVA with replication

Ixabepilone and vandetanib were tested in docetaxel-sensitive 231C cells for the capacity to activate apoptotic protein expression (values are fold of control). Drugs were tested at the concentrations that are indicated in Figure 6A.

treatment	cleaved caspase	bax/bcl2	bak/bcl2
control	1.00	1.29	0.97
	1.21	1.06	1.06
	0.82	1.31	1.20
vandetanib low	1.20	1.93	0.34
	1.63	0.88	1.63
	0.92	1.92	1.54
vandetanib high	2.30	1.93	1.46
	2.90	0.99	1.54
	1.52	2.12	1.74
ixabepilone	8.04	3.21	2.46
	9.82	5.03	2.96
	6.26	3.11	2.48
ixabepilone + vandetanib low	19.97	2.90	5.79
	13.73	4.20	3.19
	7.49	3.57	3.17
ixabepilone + vandetanib high	16.00	7.62	5.20
	19.89	5.20	6.61
	11.78	6.78	5.82

cleaved caspase-3

ANOVA Table for cleaved caspase

	DF	Sum of Squares	Mean Square	F-Value	P-Value
Column 1	5	651.008	130.202	13.204	.0002
Residual	12	118.330	9.861		

Model II estimate of between component variance: 40.114

Means Table for cleaved caspase
Effect: Column 1

	Count	Mean	Std. Dev.	Std. Err.
control	3	1.010	.190	.110
vand low	3	1.250	.380	.219
vand high	3	2.240	.690	.398
ixa	3	8.040	1.780	1.028
ixa + vand low	3	13.730	6.240	3.603
ixa + vand high	3	15.890	4.050	2.338

Comparison	mean difference	critical difference	p value
Control vs vandetanib low	0.2	5.59	0.9270
Control vs vandetanib high	1.2		0.6400
Control vs ixabepilone	7.0		0.0179
Control vs vandetanib low plus ixabepilone	12.7		0.0003
Control vs vandetanib high plus ixabepilone	14.9		<0.0001

Bax/Bcl-2 ratio

ANOVA Table for bax/bacl-2

	DF	Sum of Squares	Mean Square	F-Value	P-Value
Column 1	5	60.973	12.195	19.005	<.0001
Residual	12	7.700	.642		

Model II estimate of between component variance: 3.851

Means Table for bax/bacl-2
Effect: Column 1

	Count	Mean	Std. Dev.	Std. Err.
control	3	1.220	.139	.080
vand low	3	1.577	.603	.348
vand high	3	1.680	.605	.349
ixa	3	3.783	1.081	.624
ixa + vand low	3	3.557	.650	.375
ixa + vand high	3	6.533	1.229	.709

Comparison	mean difference	critical difference	p value
Control vs vandetanib low	0.36	1.43	0.5955
Control vs vandetanib high	0.46		0.4953
Control vs ixabepilone	2.56		0.0020
Control vs vandetanib low plus ixabepilone	2.34		0.0038
Control vs vandetanib high plus ixabepilone	5.31		<0.0001

Bak/Bcl-2 ratio

ANOVA Table for bak/bcl-2

	DF	Sum of Squares	Mean Square	F-Value	P-Value
Column 1	5	54.429	10.886	19.191	<.0001
Residual	12	6.807	.567		

Model II estimate of between component variance: 3.439

Means Table for bak/bcl-2
Effect: Column 1

	Count	Mean	Std. Dev.	Std. Err.
control	3	1.077	.116	.067
vand low	3	1.170	.720	.416
vand high	3	1.580	.144	.083
ixa	3	2.633	.283	.163
ixa + vand low	3	4.050	1.507	.870
ixa + vand high	3	5.877	.707	.408

Comparison	mean difference	critical difference	p value
Control vs vandetanib low	0.09	1.34	0.8819
Control vs vandetanib high	0.50		0.4290
Control vs ixabepilone	1.56		0.0263
Control vs vandetanib low plus ixabepilone	2.97		0.0004
Control vs vandetanib high plus ixabepilone	4.80		<0.0001

Supplementary analysis for Figure 6B: Original data and One way ANOVA with replication

Ixabepilone and vandetanib were tested in docetaxel-resistant TXT cells for the capacity to activate apoptotic protein expression (values are fold of control). Drugs were tested at the concentrations that are indicated in Figure 6B.

treatment	cleaved caspase	bax/bcl2	bak/bcl2
control	1.22	1.11	0.95
	1.01	1.10	1.06
	0.80	1.13	1.02
vandetanib low	1.30	0.46	0.98
	1.91	0.46	0.99
	0.81	1.34	1.35
vandetanib high	1.30	1.12	0.52
	1.77	0.72	1.14
	0.72	0.66	1.32
ixabepilone	7.00	4.96	2.40
	5.42	1.83	1.16
	8.79	1.76	3.06
ixabepilone + vandetanib low	10.50	16.40	17.60
	12.24	10.30	21.00
	9.21	8.15	26.30
ixabepilone + vandetanib high	13.33	10.29	25.30
	15.99	13.30	19.10
	14.66	13.20	26.40

cleaved caspase-3

ANOVA Table for cleaved caspase

	DF	Sum of Squares	Mean Square	F-Value	P-Value
Column 1	5	501.108	100.222	80.062	<.0001
Residual	12	15.022	1.252		

Model II estimate of between component variance: 32.99

Means Table for cleaved caspase
Effect: Column 1

	Count	Mean	Std. Dev.	Std. Err.
control	3	1.010	.210	.121
vand low	3	1.340	.570	.329
vand high	3	1.240	.520	.300
ixa	3	7.070	1.680	.970
ixa + vand low	3	10.650	1.510	.872
ixa + vand high	3	14.660	1.330	.768

Comparison	mean difference	critical difference	p value
Control vs vandetanib low	0.33	1.99	0.7242
Control vs vandetanib high	0.23		0.8055
Control vs ixabepilone	6.06		<0.0001
Control vs vandetanib low plus ixabepilone	9.64		<0.0001
Control vs vandetanib high plus ixabepilone	13.65		<0.0001

Bax/Bcl-2 ratio

ANOVA Table for Bax/Bcl-2

	DF	Sum of Squares	Mean Square	F-Value	P-Value
Column 1	5	454.818	90.964	21.919	<.0001
Residual	12	49.800	4.150		

Model II estimate of between component variance: 28.938

Means Table for Bax/Bcl-2
Effect: Column 1

	Count	Mean	Std. Dev.	Std. Err.
control	3	1.113	.015	.009
vand low	3	.753	.508	.293
vand high	3	.833	.250	.144
ixa	3	2.850	1.828	1.055
ixa + vand low	3	11.617	4.280	2.471
ixa + vand high	3	12.263	1.710	.987

Comparison	mean difference	critical difference	p value
Control vs vandetanib low	0.36	3.62	0.8323
Control vs vandetanib high	0.28		0.8691
Control vs ixabepilone	1.74		0.3170
Control vs vandetanib low plus ixabepilone	10.50		<0.0001
Control vs vandetanib high plus ixabepilone	11.15		<0.0001

Bak/Bcl-2 ratio

ANOVA Table for Bak/Bcl-2

	DF	Sum of Squares	Mean Square	F-Value	P-Value
Column 1	5	1821.535	364.307	60.942	<.0001
Residual	12	71.735	5.978		

Model II estimate of between component variance: 119.443

Means Table for Bak/Bcl-2
Effect: Column 1

	Count	Mean	Std. Dev.	Std. Err.
control	3	1.010	.056	.032
vand low	3	1.107	.211	.122
vand high	3	.993	.420	.242
ixa	3	2.207	.965	.557
ixa + vand low	3	21.633	4.384	2.531
ixa + vand high	3	23.600	3.936	2.272

Comparison	mean difference	critical difference	p value
Control vs vandetanib low	0.97	4.35	0.8819
Control vs vandetanib high	0.02		0.9935
Control vs ixabepilone	1.20		0.5600
Control vs vandetanib low plus ixabepilone	20.62		<0.0001
Control vs vandetanib high plus ixabepilone	22.59		<0.0001

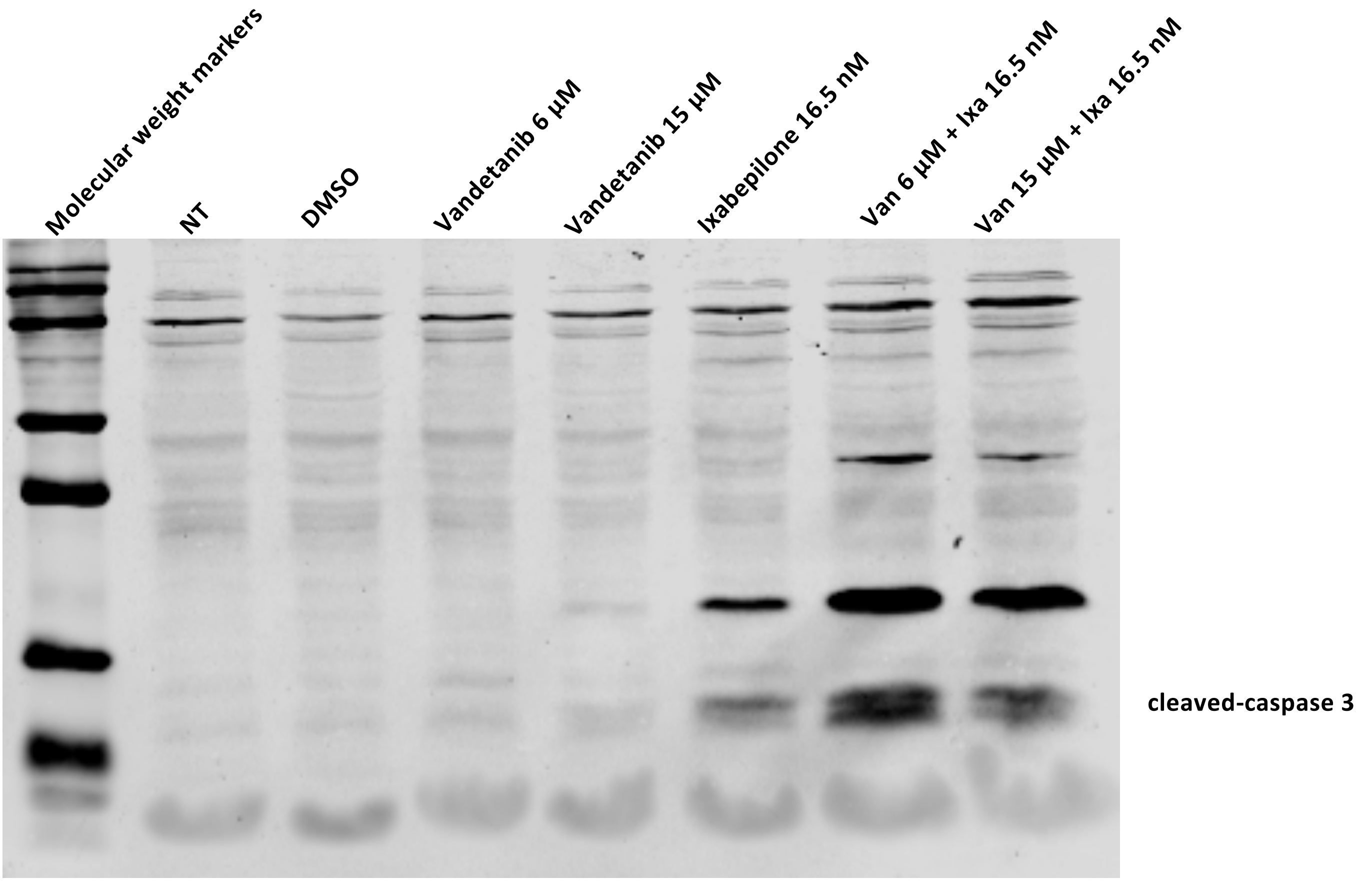

Supplementary Figure 1: Full western blots for cleaved caspase-3 immunoreactive protein in 231C cells treated with ixabepilone and vandetanib as indicated.

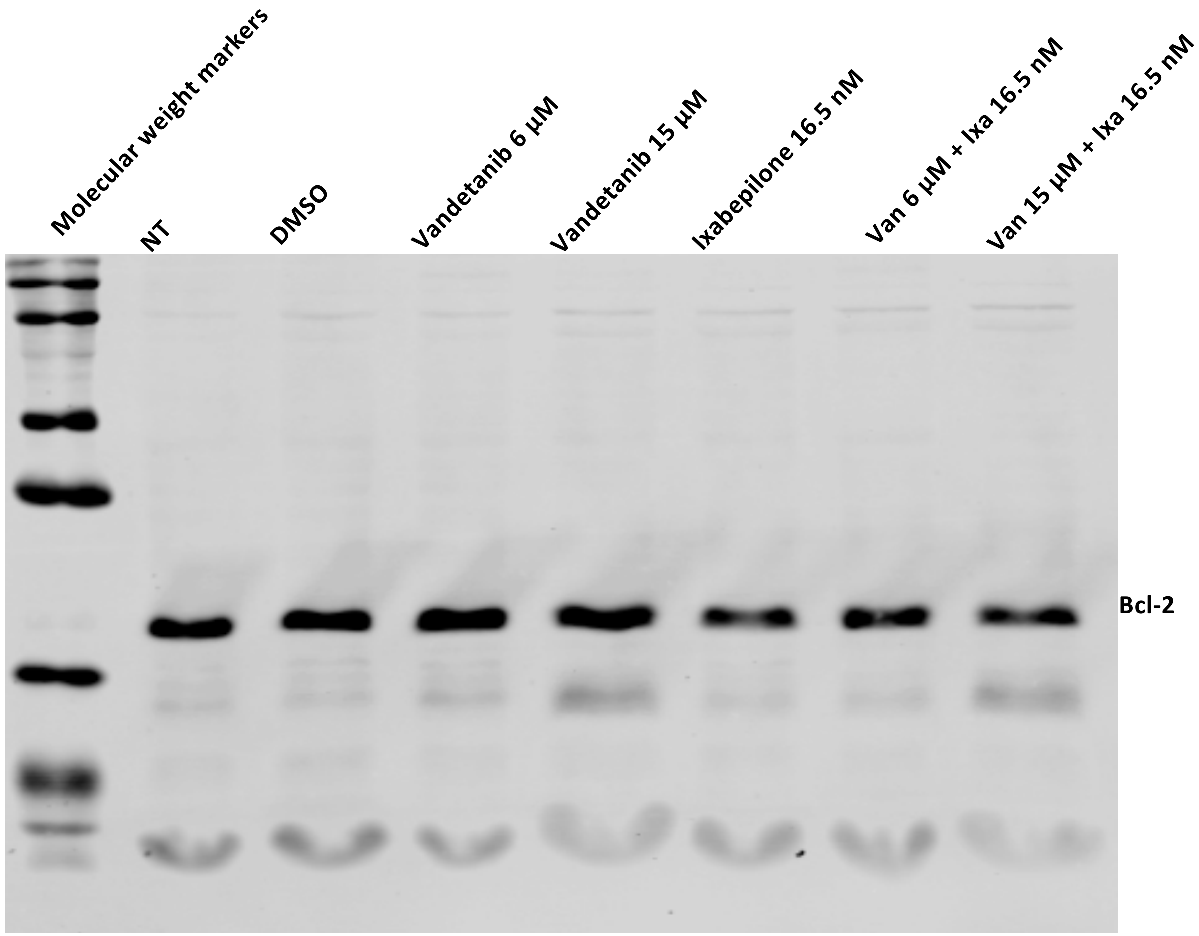

Supplementary Figure 2: Full western blots for Bcl-2 immunoreactive protein in 231C cells treated with ixabepilone and vandetanib as indicated.

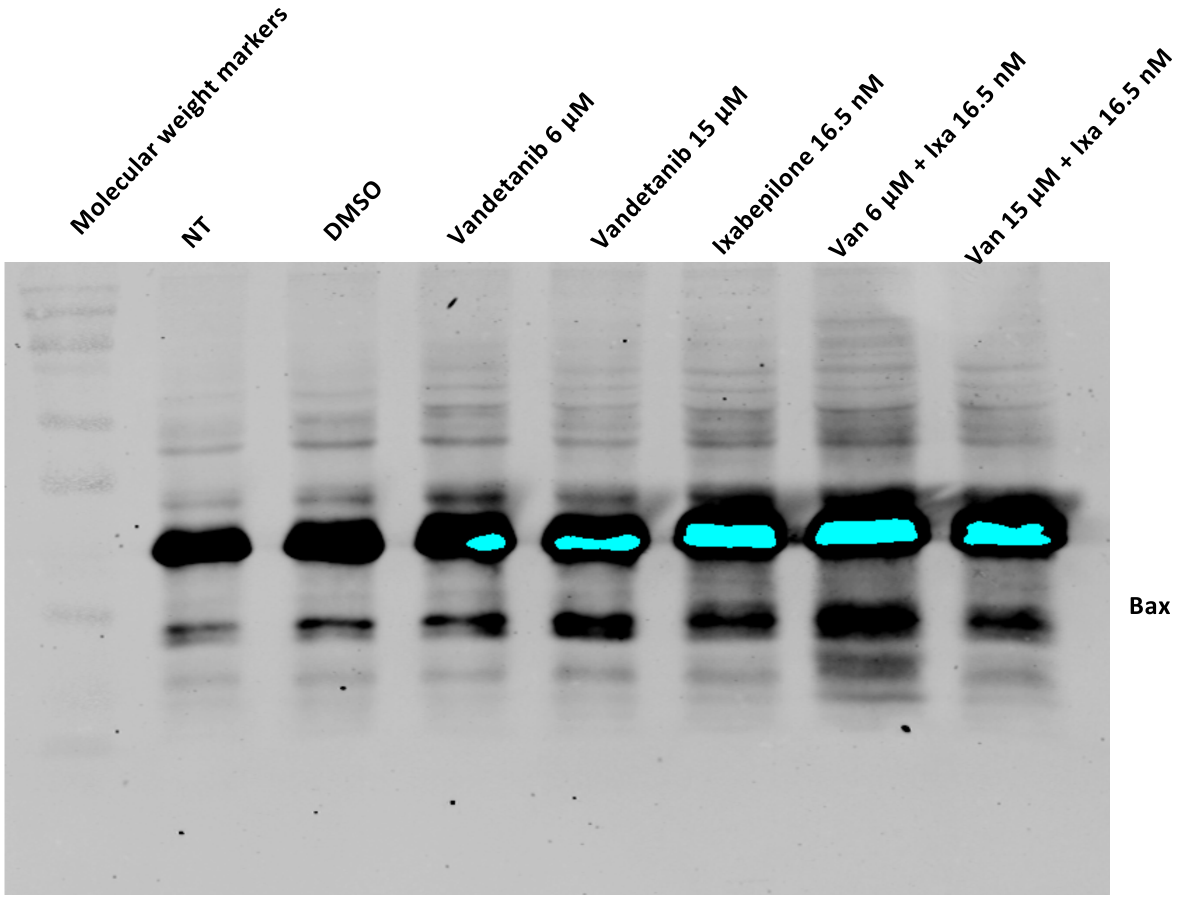

Supplementary Figure 3: Full western blots for Bax immunoreactive protein in 231C cells treated with ixabepilone and vandetanib as indicated.

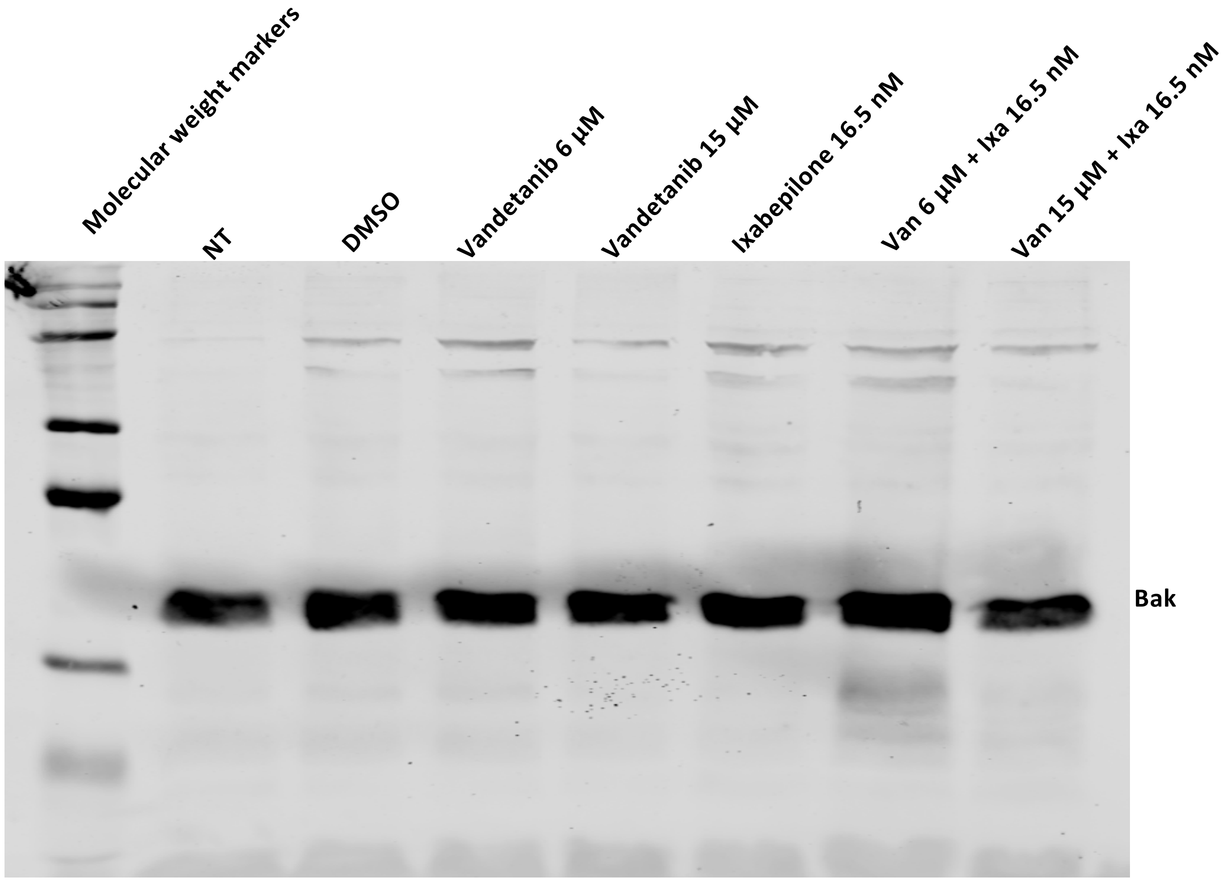

Supplementary Figure 4: Full western blots for Bak immunoreactive protein in 231C cells treated with ixabepilone and vandetanib as indicated.

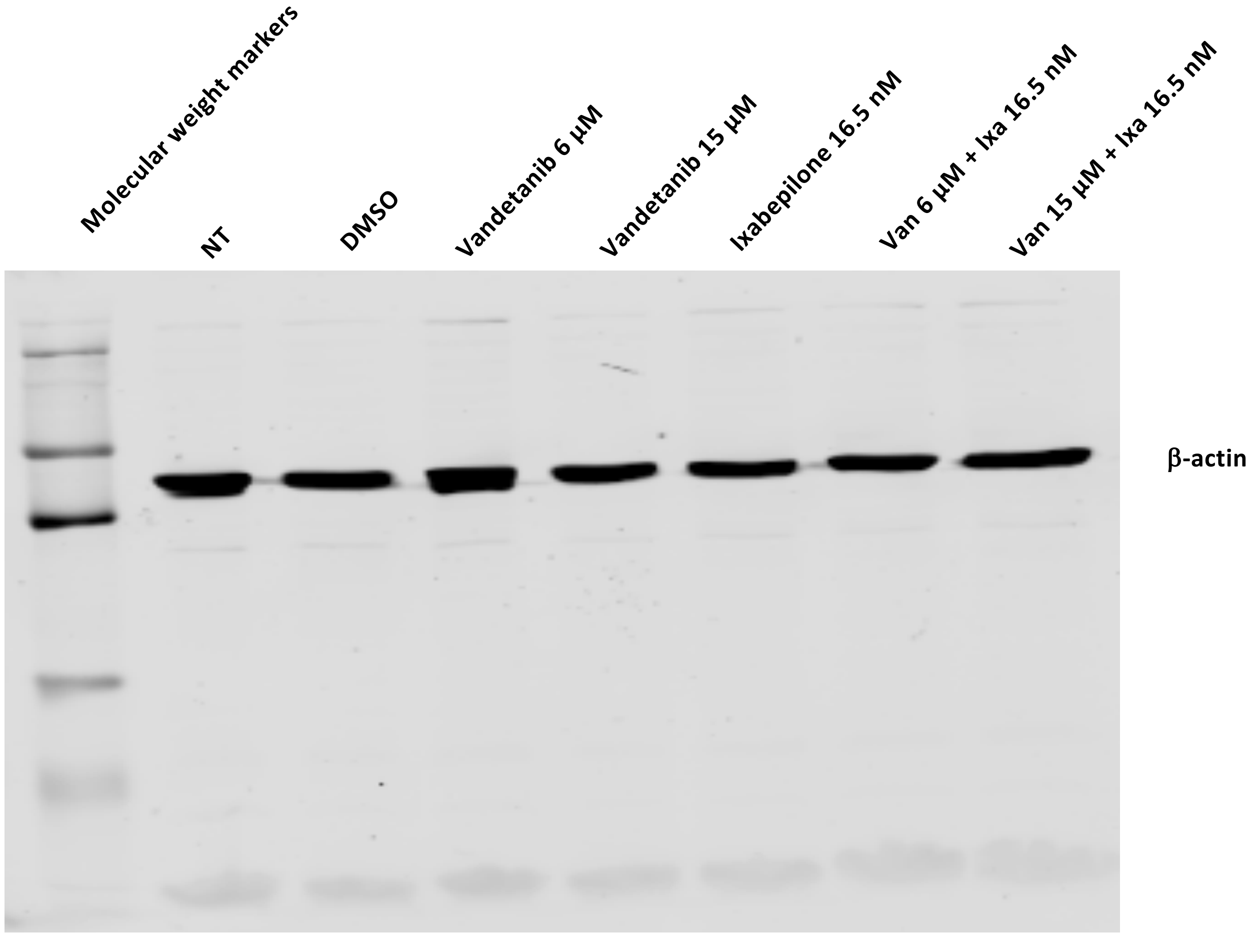

Supplementary Figure 5: Full western blots for β-actin immunoreactive protein in 231C cells treated with ixabepilone and vandetanib as indicated.

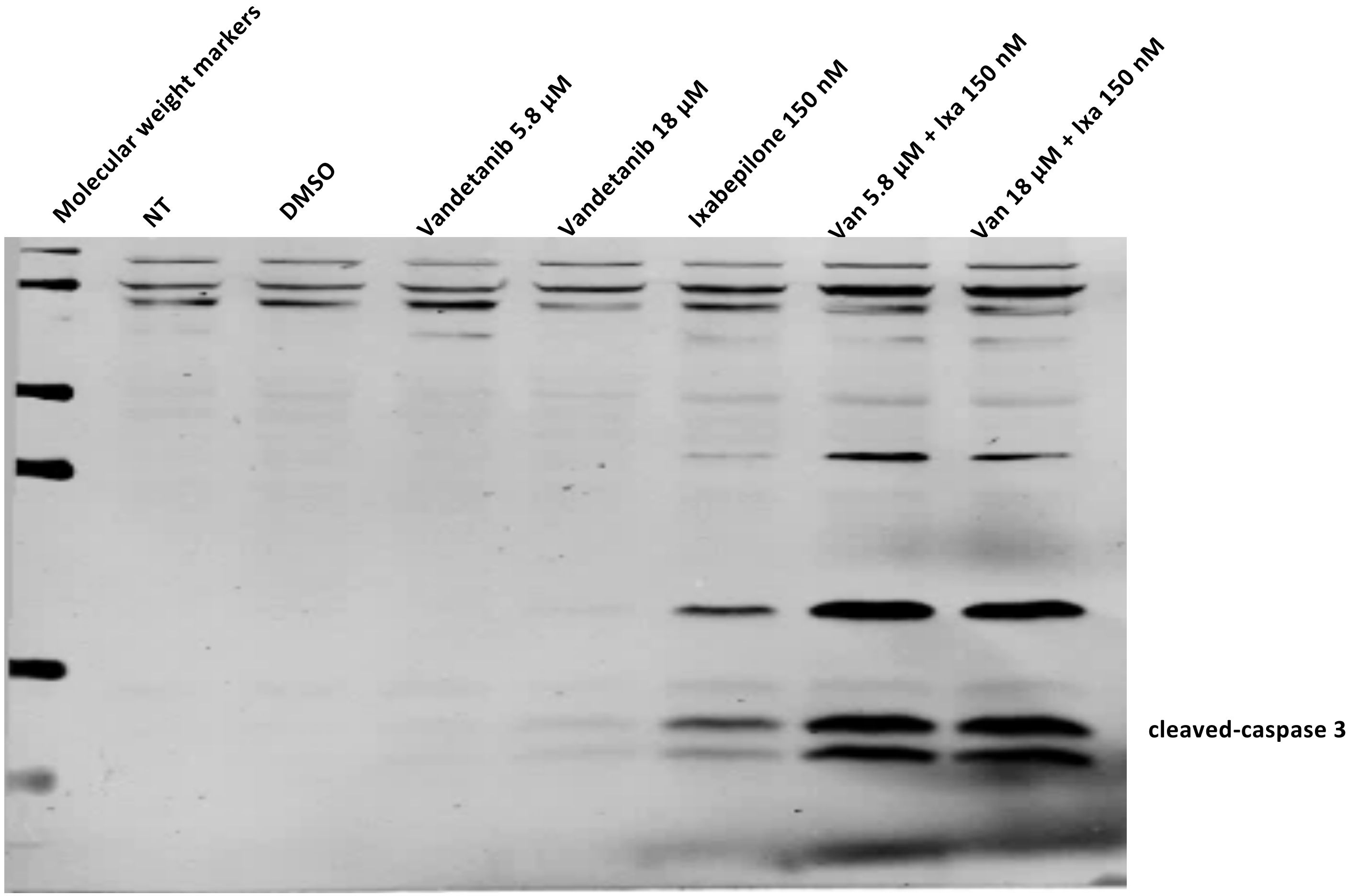

Supplementary Figure 6: Full western blots for cleaved caspase-3 immunoreactive protein in TXT cells treated with ixabepilone and vandetanib as indicated.

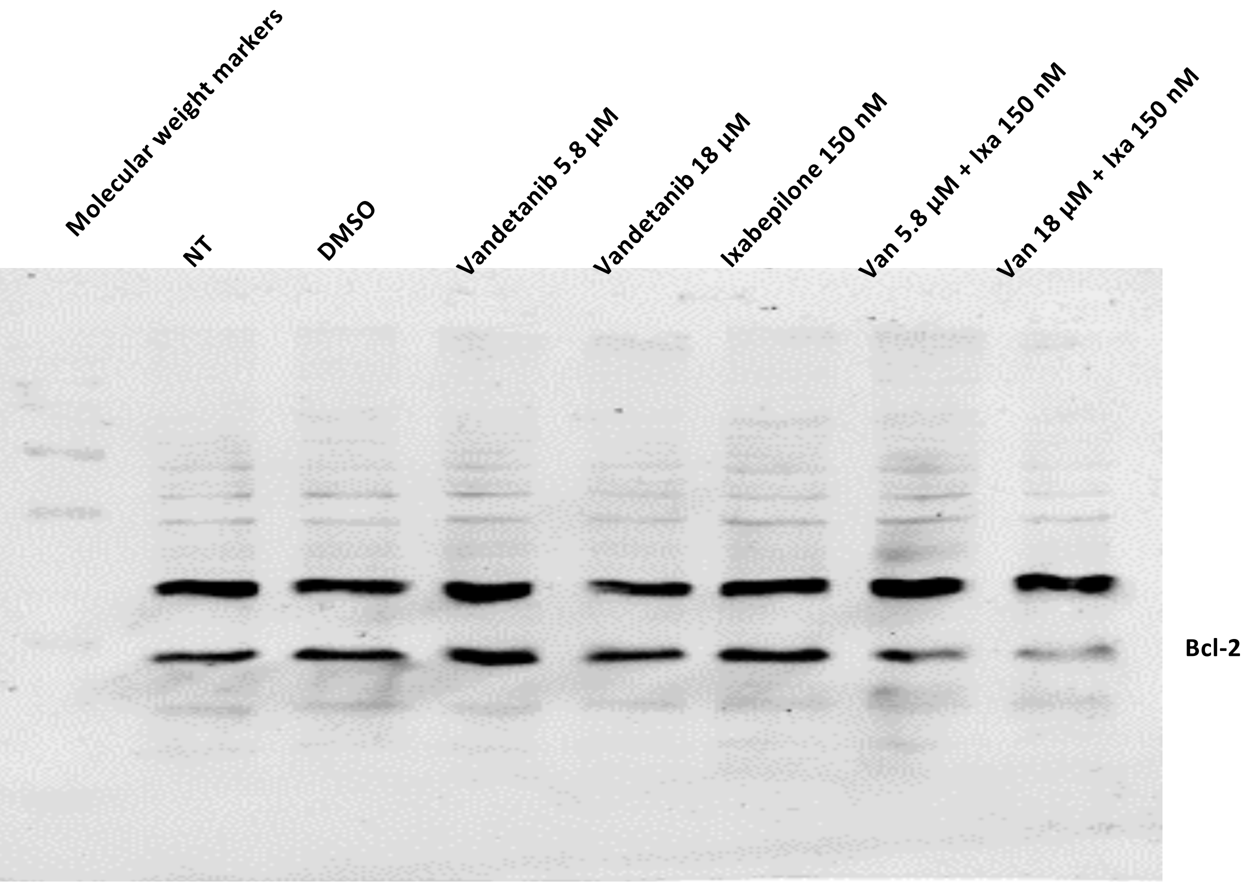

Supplementary Figure 7: Full western blots for Bcl-2 immunoreactive protein in TXT cells treated with ixabepilone and vandetanib as indicated.

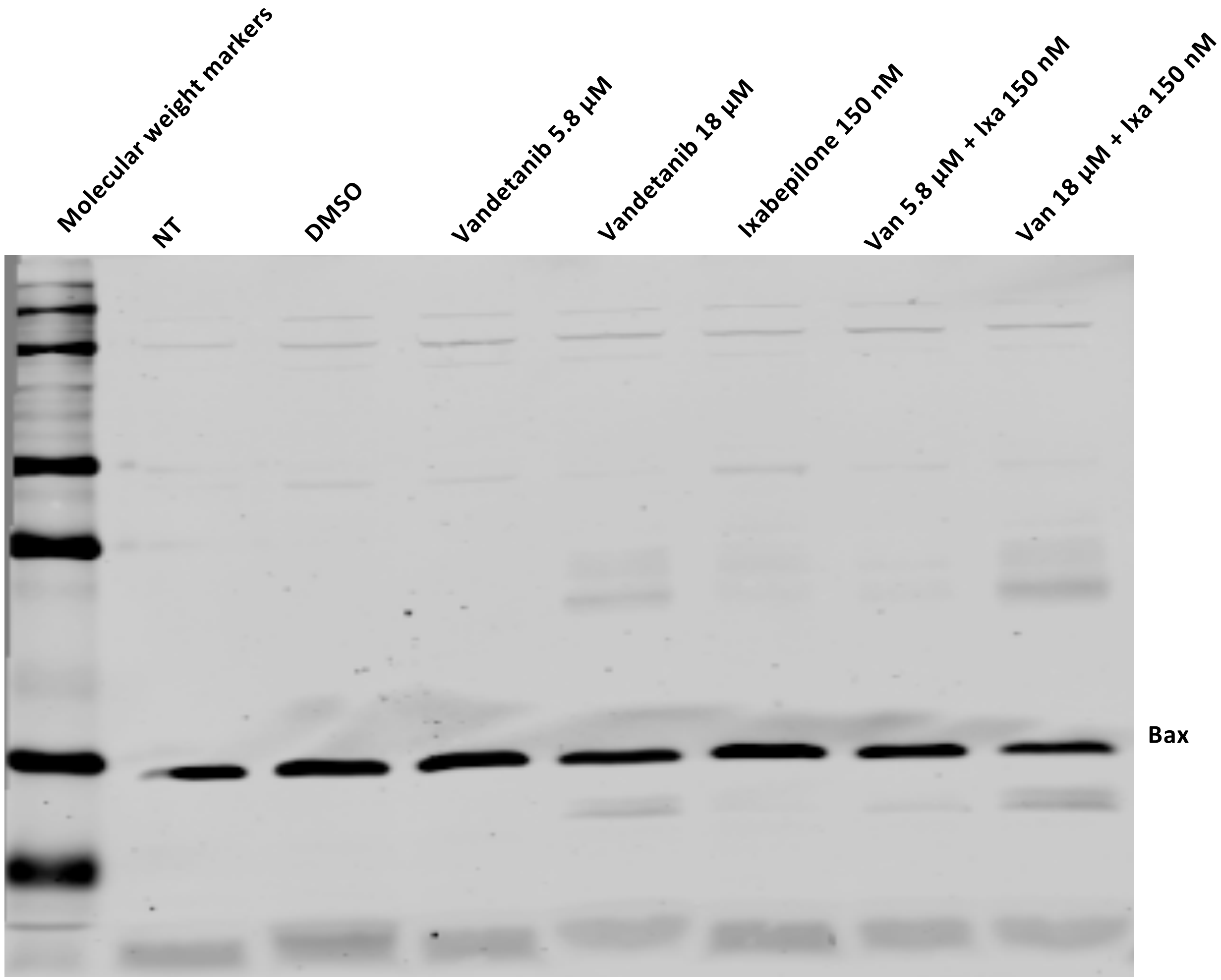

Supplementary Figure 8: Full western blots for Bax immunoreactive protein in TXT cells treated with ixabepilone and vandetanib as indicated.

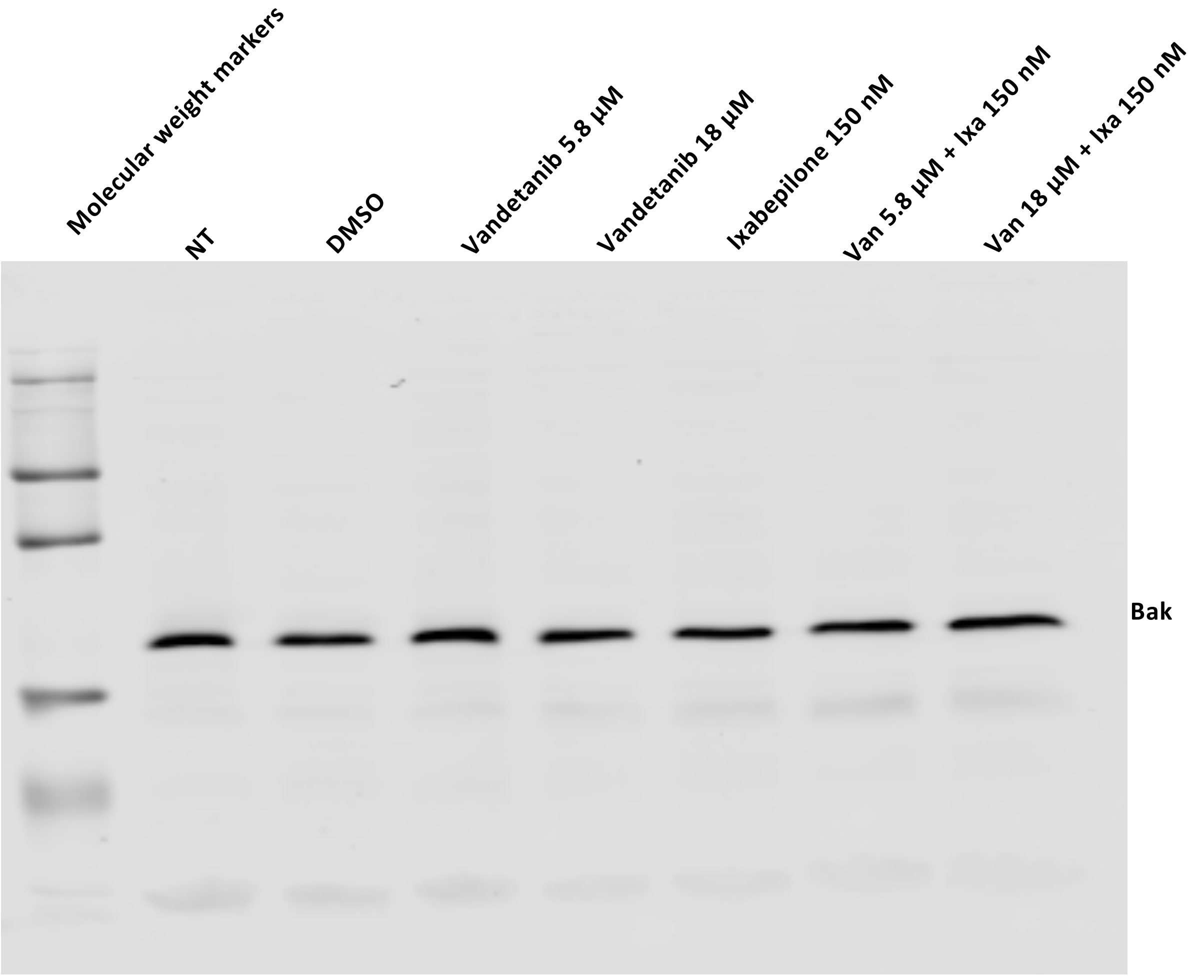

Supplementary Figure 9: Full western blots for Bak immunoreactive protein in TXT cells treated with ixabepilone and vandetanib as indicated.

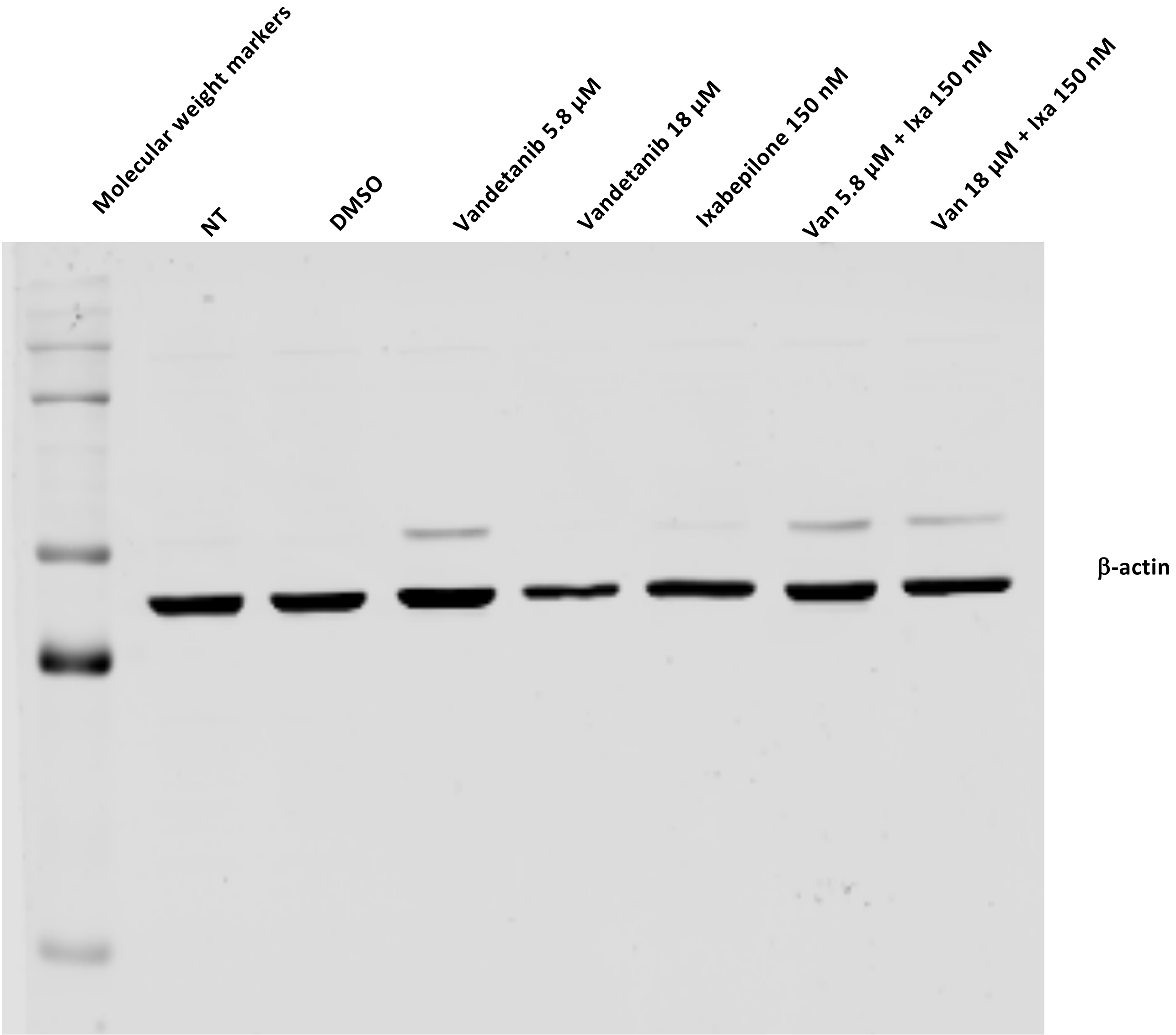

Supplementary Figure 10: Full western blots for β-actin immunoreactive protein in TXT cells treated with ixabepilone and vandetanib as indicated.
